# Toward Sustaining Web-Based Senior Center Programming Accessibility With and for Older Adult Immigrants: Community-Based Participatory Research Cross-Sectional Study

**DOI:** 10.2196/49493

**Published:** 2024-01-26

**Authors:** Connie Kim Yen Nguyen-Truong, Katherine Wuestney, Holden Leung, Chenya Chiu, Maria Park, Christina Chac, Roschelle Lynette Fritz

**Affiliations:** 1 Nursing and Systems Science Department College of Nursing in Vancouver Washington State University Vancouver, WA United States; 2 PhD in Nursing Program College of Nursing, Spokane Health Sciences Washington State University Spokane, WA United States; 3 Asian Health & Service Center Portland, OR United States

**Keywords:** Asian American, Chinese, Korean, Vietnamese, community-based participatory research, CBPR, COVID-19, health equity, immigrants, older adults, psychosocial, technology access, telehealth use, web-based senior center, mobile phone

## Abstract

**Background:**

During the COVID-19 pandemic, many community-based organizations serving Asian Americans pivoted to provide web-based care and social services. Asian American community leaders in the United States Pacific Northwest, including Asian Health & Service Center expressed that there are older immigrant adults who experienced backlash from discrimination, fear, and anxiety owing in part to anti-Asian hate and isolation, including from infection precautions. Pivoting supported staying safe from COVID-19 transmission and anti-Asian hate crimes.

**Objective:**

This study aims to examine the readiness of diverse groups of older Asian American immigrant adults (Chinese, Koreans, and Vietnamese) to use a web-based senior center, including technology access and telehealth use, and to identify the psychosocial health impacts that a web-based senior center could be positioned to meet.

**Methods:**

A community-based participatory research approach was used to conduct a cross-sectional survey study in an Asian-based health and service center in 2022. We selected surveys from the National Institutes of Health–supported PhenX Toolkit. Analyses were performed using R software.

**Results:**

There was an 88.2% (216/245) response rate. Overall, 39.8% (86/216) of participants were Chinese, 25% (54/216) were Korean, and 24.5% (53/216) were Vietnamese. There were significant group differences in mobile data plans (*P*=.0005). Most had an unlimited mobile data plan (38/86, 44% Chinese; 39/54, 72% Koreans; 25/53, 47% Vietnamese). Significant group differences existed regarding whether they started using a new electronic device to communicate with friends or family after the COVID-19 outbreak (*P=*.0005); most were Korean participants (31/54, 57%). For written text and audio or video apps, most Chinese participants used WeChat (65/85, 76%; 57/84, 68%, respectively), most Koreans used KakaoTalk (49/54, 91%; 49/54, 91%, respectively), and most Vietnamese used Facebook Messenger for written text (32/50, 64%) and Apple Face Time (33/50, 66%) or Facebook Messenger (31/50, 62%) for audio or video. Significant group differences existed regarding whether to try telehealth (*P*=.0005); most Vietnamese expressed that they would never consider it (41/53, 77%). Significant group differences existed regarding how well they were able to concentrate (*χ*^2^_2_*=*44.7; *P*<.0001); Chinese participants reported a greater inability (median 5, IQR 4-6). With regard to difficulties in life experiences (*χ^2^*_2_=51; *P*<.0001), the median was 6 (IQR 5-7) for the Vietnamese group. Significant group differences existed in having had a family/household member’s salary, hours, and contracts reduced (*P*=.0005) and having had a family/household member or friend fallen physically ill (*P*=.0005)—most Vietnamese (15/53, 28%) and Korean participants (10/53, 19%).

**Conclusions:**

To build an efficacious, web-based senior center with web-based care and social service options, more older adults need access to the internet and education about using technology-enabled communication devices. Addressing the unique psychosocial impacts of the COVID-19 pandemic on each group could improve health equity. The strength of the participating older adults was observed and honored.

## Introduction

### Background

During the COVID-19 pandemic, many community-based organizations (CBOs), such as culturally based health and social service centers quickly pivoted to provide web-based services to maintain contact with clients. Although the pivot to web-based contact helped to maintain care and social services, questions remain about how to best provide web-based care and social services and whether older adults can access care and services in a meaningful way. The sustainability of web-based care and social services is important because of reports by older adults that they continue to experience anti-Asian hate [1,2] and isolation [1]. During the COVID-19 pandemic, many Asian Americans avoid leaving their home to go to public places such as grocery stores, church, and school, and many have not talked with a health care provider or mental health professional about their feelings of isolation [3]. Providing web-based services can address both safety and isolation concerns; however, it is important for CBOs serving older Asian Americans to understand how they engage with technology and which devices and platforms they commonly use. Our community and academic partnership studied these issues at the request of a CBO serving Asian Americans in the Pacific Northwest. Findings reflect a drive toward health equity and responsiveness to community-identified priorities for sustaining and growing web-based social and health services after the COVID-19 pandemic. We intentionally disaggregated group data into granular, within group–specific data to address concerns expressed in the extant literature [2,4,5] and by CBOs that aggregated Asian American data are not always helpful at an actionable community level for countering systemic issues and for advancing health equity ideals.

Many Asian American CBOs serving older adults reported escalated racial discrimination during the COVID-19 pandemic, such as hate crimes or microaggressions [6]. Asian Americans experienced aggravated physical and mental health problems or violence [4]. Many were afraid to seek care because of anti-Asian xenophobia [5]. Older Asian American immigrants continue to be particularly vulnerable when they leave home owing to hate crimes against Asian individuals, with great adverse effects experienced by older adults who are undocumented, facing poverty, and having limited English proficiency [5]. Between March 2020 and April 2023, Stop AAPI Hate received 17,804 reports of hate incidents, including verbal harassment, shunning, physical assault, civil rights violations, harassment via the web, and more [1]. Asian Americans experienced psychological distress, stress, and depression during the COVID-19 pandemic. Southeast Asian individuals experienced more psychological distress than White (not Hispanic) individuals [7]. Chinese and Vietnamese reported that racial and ethnic discrimination and violence against their population led to feelings of stress and depression, and some reported being treated unfairly because of their race and ethnicity [3]. Furthermore, Koreans with preexisting chronic diseases were heavily affected, thus experiencing worse health outcomes [8]. Despite these known discriminations amid the COVID-19 pandemic, studying mental health among Asian Americans, particularly among Asian subgroups, was not prioritized in the United States [7]. Web-based care can be a necessary response to address continuity in delivering care and social services for constituents at risk for infection and criminal victimization.

Web-based care was a part of the pivot during the COVID-19 pandemic; however, this was isolating to many older Asian American immigrants [6]. Older adult users in the general population increasingly integrate technology and mobile devices into their daily lives [9], but this is not necessarily true for older Asian Americans. A study including older White Canadians showed that they were primarily concerned with avoiding the virus and with health care efficiencies [1] that web-based services can address, whereas older Korean immigrants were primarily worried about autonomy, technology dependence, and the burden of learning a new technology for engaging in social and health services [10]. Such worries, along with more broadly reported concerns by older adults about needing to be technology savvy and wanting in-person physical health exams [11] are not easily mitigated with web-based services. Despite these findings, in a national study of 40 CBOs serving Asian Americans, researchers found that technology was a connector for organizations [6]. Thus, understanding how to integrate technology in a meaningful way is important for successfully sustaining web-based contact with clients. Many resilient organizations have reflected on their commitment to serve communities with pride by adapting and preparing to face future crises [6].

### Community Context

Asian American community leaders at the Asian Health & Service Center (AHSC) in Oregon in the United States Pacific Northwest expressed concerns that there are older Asian American immigrant adults who experienced backlash regarding discrimination, fear, and anxiety in part from anti-Asian hate and isolation, including from social distancing for infection protection since the COVID-19 outbreak. AHSC is a culturally diverse, nonprofit CBO and a trusted source for health care and social services [12]. Most clients are older Chinese, Korean, and Vietnamese immigrants [1]. The chief executive director reported that they required a fast pivot to use more technology owing to concerns expressed by older Asian American immigrant adults. This pivot included training staff to deliver health care and social services remotely (ie, distance). COVID-19 Asian response teams were created that consisted of community health workers (CHWs) and behavioral health counselors. Health care and social services were delivered via audio and video calls while attempting to maintain the AHSC holistic health care and social services model of social engagement, public health information, and support for health needs. There is a crucial need to engage to inform rebuilding as a web-based senior center after COVID-19 with a web-based care and social services option. AHSC community leaders identified priorities based on expressed concerns, and this included engaging culturally diverse, older Asian American immigrant adult clients by centering their voice, learning about their technology access and telehealth use to extend reach in client support and mental health counseling, and uplifting a dedicated community workforce of culturally diverse and multilingual CHWs for web-based outreach and care of older Asian American immigrant adults to advance health equity. AHSC community leaders raised that conducting a survey study can be a step in centering the voice and engaging the participation of older Asian American immigrant adults by clarifying what is meant by web-based care.

### Community Engagement to Advance Health Equity

Community engagement is essential to advance health equity. Academic and community researchers should comprehensively embed methods of community-based participatory research (CBPR) that is action oriented into the design of research studies [13]. There is a need to fully engage communities in community-involved care settings to ensure sustainability in the context of direct application to real-world care delivery [13]. Community engagement in scientific design and procedures is important for collaborative research decision-making based on a shared working understanding [1]. Community engagement through CBPR and citizen science, where participatory action drives research direction for sustainability in population health science, is important [1,14]. Authentic intentionality for an inclusive collaboration partnership needs to include conceptualization, design, implementation, and dissemination. The Community Connected Health initiative set forth by the White House Office of Science and Technology Policy underscored the need to work with communities. Emphasis is placed on CBOs to prioritize their technological needs and goals while integrating strengths and keeping the end users in mind while designing and have support for a representative and diverse health technology workforce [15]. Furthermore, a need exists for thoughtful approaches to equity and inclusion in collecting and using data and for organizations to be involved in community-based health care delivery through actionable data [15]. We built upon a long-standing, cross-sector, and trusted community-academic partnership between AHSC and a public Washington State University (WSU) College of Nursing since 2015. As a community and academic partnership, we conducted previous CBPR studies regarding capacity building on health-assistive smart home monitoring technology adoption and perceptions about smart home adoption by older Asian American immigrant adults, including Chinese, Koreans, and Vietnamese. Details were reported elsewhere [16,17].

### This CBPR Study

CBO leaders organically drove the purpose, aims, and design for this study in partnership with a nursing science research team. The aims they identified for this CBPR cross-sectional survey study were to explore two domains: (1) examine the readiness of diverse groups of Asian American immigrant older adults (Chinese, Koreans, and Vietnamese) to use a web-based senior center, including technology access and telehealth, and (2) identify the psychosocial health impacts of older Asian American immigrants among Chinese, Korean, and Vietnamese groups that a web-based senior center could be positioned to meet. As a step to understand the potential sustainability of web-based social and health services, we investigated the behaviors and attitudes toward the internet; access to the internet and associated devices; experiences and attitudes toward telehealth; and psychosocial impacts, including needs and effects of the COVID-19 outbreak on diverse Asian American immigrant groups of older adults (Chinese, Koreans, and Vietnamese) in the United States Pacific Northwest. Findings may inform future studies in maintaining and growing web-based senior centers with a web-based care option for a culturally diverse, nonprofit, Asian-based health and social service center.

## Methods

### CBPR Cross-Sectional Survey Study Design

We used a CBPR approach to design, implement, and interpret this cross-sectional survey study and used the principles of mutual trust, rapport, respect, learning, and mentoring [18]. CBPR included equitable involvement of diverse partners throughout the research and dissemination process [18]. Our cross-sector partnership was culturally diverse, multilingual, and multidisciplinary. WSU College of Nursing academic nurse researchers included the principal investigator (PI) with a Vietnamese and Guamanian Micronesian Islander background, specialty in CBPR with immigrants and marginalized communities, and health equity in health-assistive technology adoption; co-PI with a White and Native American background and smart home health-assistive monitoring and informatics specialty; and a statistician with a White background and history in data analysis and management and smart home health-assistive monitoring. AHSC community partners included the chief executive director with a Chinese background and experience in social work and immigrant community health; 3 program managers in community health including the senior program manager with a Korean background and specialty in aging and disability, community program manager with a Chinese background and public health management and policy specialty, and community health project manager with a Chinese background and public health specialty; and 4 CHWs with a Chinese, Korean, or Vietnamese background and specialties in psychology, communication disorders, and sciences; education; fine arts; or health promotion and health behavior.

We used surveys from the National Institutes of Health–supported PhenX Toolkit that included the COVID-19 Technology Accessibility Survey (for technology access), Technology Telehealth Use, and Psychosocial Impact of COVID-19 Survey [19]. In addition, the PI, co-PI, and AHSC chief executive director codeveloped the items about written and audio or video communication apps, internet service provider, mobile phone use, mobile data plan, and access to the internet via a mobile phone (ie, technology access). The PI and co-PI consulted with a biostatistician and discussed with the nurse researcher statistician regarding the selection of items and technical functionality. The chief executive director and 3 program managers at AHSC reviewed and pretested the survey (Multimedia Appendices 1 and 2) for face validity and technical functionality. AHSC community partners discussed among themselves about meaningful interpretation and discussed with academic nurse researchers on a regular basis throughout the research process. AHSC community partners spoke English and Chinese Cantonese, Chinese Mandarin, Korean, or Vietnamese and assisted the academic nurse researchers with outreach, recruitment, and interpretation. This aligns with the AHSC holistic health care and social services model, which provides cultural and linguistic interpretation in the context of a real-world health and social service setting [12]. The study was conducted at the AHSC in the United States Pacific Northwest between March 2022 and April 2022.

### Ethical Considerations

This study underwent a limited review and received a certificate of exemption from full board review by the WSU Human Research Protection Program (18816). Each participant received a shopping gift card worth US $10 (eg, grocery) upon completion that honored and thanked them.

### Measurements

As of October 29, 2020, at the beginning of the CBPR design, the PI reviewed 94 COVID-19 survey protocols that were made publicly available for use, the PhenX Toolkit by the trans–National Institutes of Health working group, that consisted of the National Institute on Aging and the Office of Behavioral and Social Sciences Research [20]. Owing to the urgency of need for COVID-19–specific survey measurements at the time, these items did not undergo the same level of standardization, harmonization, or psychometric testing as per the PhenX consensus process [20]. Our academic and community partnership discussed, selected, consulted, and pretested the survey as described previously in the *CBPR Cross-Sectional Survey Study Design* section. Therefore, each item incorporated in the survey was treated as its own variable, rather than contributing to the measurement scales. The survey consisted of the following: sociodemographic and background items from our previous CBPR [17]; technology access items from the National Institute on Aging Alzheimer’s Disease Research Centers and Levey [21] and from the items codeveloped by the PI, co-PI, and AHSC chief executive director; telehealth use items from the Institute on Aging at the University of Florida [22]; and psychosocial health impact items from the National Institute of Mental Health Intramural Research Program [23]. Of the technology access items, we incorporated our codeveloped items as described previously. We used the secure and password-protected WSU Qualtrics web-based platform, formatted the survey, and entered the participant responses.

### Participants, Recruitment, and Data Collection

Overall, 7 trained program managers and CHWs at AHSC reached out to clients from the AHSC registry and used a script to provide oral information about the study primarily via telephone, with some in-person communication. The script contained information similar to the consent form that included the study purpose, investigators, eligibility, voluntary participation, procedures, shopping gift card for completion, and contact information. If an individual expressed interest, an AHSC community partner referred them to the web-based Qualtrics site that has the combined consent form, eligibility, and survey. Through the consent form, individuals were informed about the study purpose and investigators. Only a unique study number will be used to follow up for providing a shopping gift card, and it will be accessible to community-academic research partnership. Responses will be entered into the secure Qualtrics web-based platform and retained for 3 years. The survey takes approximately 30 minutes. The participants were also informed about the potential for risks, such as emotional discomfort, feeling of embarrassment, or loss of privacy if the participant chooses to have interpretation assistance. Individuals were asked to participate in the study if they were eligible and complete the survey. Participants could choose either Chinese Cantonese, Chinese Mandarin, Korean, or Vietnamese interpretation assistance from an AHSC community partner as they completed the survey. This convenient and purposive sample included a total of 216 individuals who identified as an Asian American immigrant and were aged ≥60 years.

### Data Analysis

All statistical analyses were performed using R (version 4.2.0) [24] and RStudio (version 2022.7.1) [25], with the *tidyverse* (version 1.3.1) [26], *arsenal* (version 3.6.3) [27], *labelled* (version 2.9.1) [28], and *psych* (version 2.2.5) [29] packages. We analyzed the data of older Asian American immigrants as a whole and as disaggregated data that are stratified by race and ethnicity. Of the 216 older Asian American immigrant adults, a subtotal was 193 (89.4%) across Chinese, Korean, and Vietnamese groups. There was low participation of Taiwanese and multiracial individuals, and a participant reported as being Asian and having a different ethnicity than listed previously; therefore, we described meaningful interpretation of group-wide comparisons across and among Chinese, Korean, and Vietnamese groups alongside the total group of older Asian American immigrant adults. For the purpose of this paper about learnings from a real-world CBPR survey study, we decided not to combine groups with low participation into less meaningful aggregated data. This also protects the privacy of these individual analyses. Frequencies and percentages were reported for categorical variables, ordinal variables, and variables with select-all response categories (ie, >1 response). Means (SDs) were reported for continuous variables, and medians (IQRs) were reported for continuous and ordinal variables. We used Bonferroni correction to maintain a cross-study, family-wise error rate of 0.05; thus, α=.0008 was the cutoff for statistical significance among Chinese, Korean, and Vietnamese groups. Therefore, all *P* values were reported to 4 decimals to be specific and align with a meaningful data analysis, and we reported data at a granular level to align with disaggregated data science. For mutually exclusive categorical variables, Fishers exact tests were performed to examine for any group differences and the variables under consideration (*P*=.0008). We also reported *χ*^2^ (*df*) and *P* values for continuous and ordinal variables from Kruskal-Wallis rank sum tests.

We maintained a health equity lens as a community-academic partnership. The PI and statistician discussed the data and data analysis outputs first, and then with the co-PI, and this informed a culturally responsive discussion with AHSC community partners with regard to the observed response patterns. The statistician maintained field note records that captured reflexivity where we discussed potential bias as a community-academic partnership. Analytics and outputs reflected the insights gained from these discussions, contributing to the reflexive nature of the study. These records were reviewed by the PI and discussed within the community-academic partnership. Such discussions and record keeping promoted communication transparency as a way to address potential bias. We achieved meaningful data interpretation by being responsive to community partners.

## Results

### Overview

In total, AHSC community partners reached out to 245 older Asian immigrant adult clients, of whom 25 (10.2%) declined to participate in the study. Reasons for not participating included the survey length and not having experience with telehealth. The length of time to complete the survey was 60 minutes. After the statistician performed initial screening for duplicate or erroneous entries, then we discussed as a community-academic partnership and 88.2% (216/245) of the survey responses were retained for data analysis—response rate: 216/245, 88.2% and completion rate: 216/216, 100% (ie, started and completed the survey). The completeness rate (ie, no missing responses and completed answering the survey items) was approximately 93.9% (203/216). Absolute and total numbers are shown in all tables.

### Sociodemographics and Background of Participants

In total, there were 216 older Asian American immigrant adults. Overall, 39.8% (86/216) identified as Chinese, 25% (54/216) as Korean, 24.5% (53/216) as Vietnamese, 6.9% (15/216) as Taiwanese, and 3.2% (7/216) as multiracial, and 0.5% (1/216) reported as having a different Asian ethnicity than listed previously. There were 89.4% (193/216) participants across older Chinese, Korean, and Vietnamese immigrant adults. Most Chinese (34/86, 40%) and Korean (28/54, 52%) participants had postsecondary education, and most Vietnamese participants (27/53, 51%) graduated from high school. Of the Chinese, Korean, and Vietnamese participants, 60.6% (117/193) reported <US $15,000 as total household income before taxes, of which 59% (51/86) is Chinese, 56% (30/54) is Korean, and 68% (36/53) is Vietnamese. Overall, 97.9% (189/193) of participants have a regular place of care for nonemergency health care services. Furthermore, 95% (82/86) of the Chinese participants and all Korean (54/54, 100%) and Vietnamese (53/53, 100%) participants reported having a regular place of care.

Multimedia Appendix 3 summarizes the sociodemographics and background characteristics of older Asian American immigrant adults and Chinese, Korean, and Vietnamese groups.

### Behaviors, Attitudes, and Access to the Internet and Internet-Enabled Devices

Table 1 summarizes the behaviors, attitudes, and access to the internet and internet-enabled devices of older Asian American immigrant adults and Chinese, Korean, and Vietnamese groups. Overall, 13% (7/53) of Vietnamese and 2% (2/86) of Chinese participants reported not having a mobile phone at all. In total, most participants (208/216, 96.3% older Asian American immigrants; 185/193, 95.9% across Chinese, Koreans, and Vietnamese) reported that they have or have access to a smartphone or tablet; 97% (83/86) of Chinese participants, 100% (54/54) of Korean participants, and 91% (48/53) of Vietnamese participants reported access. Less than half (97/216, 44.9% older Asian American immigrants; 85/193, 44% across Chinese, Koreans, and Vietnamese) of the participants have or have access to a PC (either desktop or laptop); 37% (32/86) of Chinese participants, 57% (31/54) of Korean participants, and 42% (22/53) of Vietnamese participants reported access. In total, most participants (160/215, 74.4% older Asian American immigrants; 144/192, 75% across Chinese, Koreans, and Vietnamese) have a national internet service provider; 71% (60/85) of Chinese participants, 82% (44/54) of Korean participants, and 76% (40/53) of Vietnamese participants have a national internet service provider. Some participants (22/215, 10.2% older Asian American immigrants; 20/192, 10.4% across Chinese, Koreans, and Vietnamese) have no internet service provider; 11% (9/85) of Chinese participants, 7% (4/54) of Korean participants, and 13% (7/53) of Vietnamese participants have no internet service provider. Most participants have an unlimited mobile data plan (116/216, 53.7% older Asian American immigrants; 102/193, 52.8% across Chinese, Koreans, and Vietnamese); 44% (38/86) of Chinese participants, 72% (39/54) of Korean participants, and 47% (25/53) of Vietnamese participants have an unlimited mobile data plan. However, there was a statistically significant difference among Chinese, Korean, and Vietnamese groups (*P*=.0005), with Korean participants reporting having unlimited data at a much higher rate (39/54, 72%) than Chinese participants (38/86, 44%) or Vietnamese participants (25/53, 47%). There were also significant differences among groups (*P*=.0005) about having started using a new electronic device to communicate with friends and family after the COVID-19 outbreak with most being Korean participants (31/54, 57%) followed by Chinese participants (15/86, 17%) and a Vietnamese (1/53, 2%) participant. There were no significant differences among groups with regard to technology savvy responses (*χ*^2^_2_=3.2; *P*=.202). Overall, very few participants (9/216, 4.2% older Asian American immigrants; 7/193, 3.6% across Chinese, Koreans, and Vietnamese) perceived themselves to be very technology savvy. Most participants perceived themselves to be only a little technology savvy (76/216, 35.2% older Asian American immigrants; 67/193, 34.7% across Chinese, Koreans, and Vietnamese; 26/86, 30% Chinese; 17/54, 32% Koreans; 24/53, 45% Vietnamese) or not at all (93/216, 43.1% older Asian American immigrants; 82/193, 42.5% across Chinese, Koreans, and Vietnamese; 41/86, 48% Chinese; 25/54, 46% Koreans; 16/53, 30% Vietnamese).

**Table 1:** Behaviors, attitudes, and access to the internet and internet-enabled devices of older Asian American immigrant adults and Chinese, Korean, and Vietnamese groups.

Variables		Total older Asian American immigrants (n=216)^a^	Subtotal across older Chinese, Korean, and Vietnamese immigrants (n=193)^b^	Chinese immigrants (n=86)^c^	Korean immigrants (n=54)^c^	Vietnamese immigrants (n=53)^c^	*P* value		Chi-square (*df*)^d^	
Does not have a mobile phone, n (%)		10 (4.6)	9 (4.7)	2 (2)	0 (0)	7 (13)	.003^e^		N/A^f^	
Has a smartphone or tablet or is able to access one, n (%)		208 (96.3)	185 (95.9)	83 (97)	54 (100)	48 (91)	.048^e^		N/A	
Has a PC (desktop or laptop) or is able to access one, n (%)		97 (44.9)	85 (44)	32 (37)	31 (57)	22 (42)	.0565^e^		N/A	
Has access to other internet-enabled device (eg, smartwatch, smart home device, or television), n (%)		15 (6.9)	13 (6.7)	8 (9)	3 (6)	2 (4)	.4213^e^		N/A	
**Who is your internet provider? (multiple responses)^g^, n (%)**								.1794^e^		N/A
	National internet service provider	160 (74.4)	144 (74.6)	60 (71)	44 (82)	40 (76)				
	Regional or local internet service provider	13 (6)	12 (6.2)	6 (7)	5 (9)	1 (2)				
	Mobile phone	7 (3.3)	6 (3.1)	5 (6)	1 (2)	0 (0)				
	National internet service provider and mobile phone	2 (0.9)	1 (0.5)	1 (1)	0 (0)	0 (0)				
	Regional or local internet service provider and mobile phone	1 (0.5)^a^	0 (0)	0 (0)	0 (0)	0 (0)				
	Specified an internet provider different from abovementioned ones	2 (0.9)	1 (0.5)	0 (0)	0 (0)	1 (2)				
	Not sure	8 (3.7)	8 (4.2)	4 (5)	0 (0)	4 (8)				
	None	22 (10.2)	20 (10.4)	9 (11)	4 (7)	7 (13)				
**Mobile data plan type, n (%)**								.0005^e^		N/A
	Capped or limited plan	10 (4.6)	9 (4.7)	6 (7)	1 (2)	2 (4)				
	Capped or limited plan amount unsure	39 (18.1)	36 (18.7)	14 (16)	6 (11)	16 (30)				
	Not applicable (ie, no mobile phone)	10 (4.6)	9 (4.7)	2 (2)	0 (0)	7 (13)				
	None	4 (1.9)	4 (2.1)	3 (4)	0 (0)	1 (2)				
	Unlimited	116 (53.7)	102 (52.8)	38 (44)	39 (72)	25 (47)				
	Unsure about plan type	37 (17.1)	33 (17.1)	23 (27)	8 (15)	2 (4)				
**Do you consider yourself to be technology savvy?**								.202^d^		3.2 (2)
	Score, median (IQR)	2 (1-2)	2 (1-2)	2 (1-2)	2 (1-2)	2 (1-2)				
	Not at all, n (%)	93 (43.1)	82 (42.5)	41 (48)	25 (46)	16 (30)				
	A little, n (%)	76 (35.2)	67 (34.7)	26 (30)	17 (32)	24 (45)				
	Somewhat so, n (%)	38 (17.6)	37 (19.2)	16 (19)	12 (22)	9 (17)				
	Very much so, n (%)	9 (4.2)	7 (3.6)	3 (4)	0 (0)	4 (8)				
**Overall, how confident do you feel using computers, smartphones, or other electronic devices to do the things you need to do online?**								.4224^d^		1.7 (2)
	Score, median (IQR)	2 (1-3)	2 (1-3)	2 (1-3)	2 (1-2.8)	2 (1-3)				
	Not at all confident, n (%)	89 (41.2)	80 (41.5)	34 (40)	26 (48)	20 (38)				
	Only a little confident, n (%)	66 (30.6)	57 (29.5)	27 (31)	14 (26)	16 (30)				
	Somewhat confident, n (%)	50 (23.1)	47 (24.4)	21 (24)	14 (26)	12 (23)				
	Very confident, n (%)	11 (5.1)	9 (4.7)	4 (5)	0 (0)	5 (9)				
Have you started using a new electronic device to communicate with friends and family after the COVID-19 outbreak? (yes), n (%)		52 (24.1)	47 (24.4)	15 (17)	31 (57)	1 (2)	.0005^e^		N/A	
**Before the COVID-19 outbreak, would you say technology has had a mostly positive effect on our society or a mostly negative effect on our society^h^?**								.0221^d^		7.6 (2)
	Score, median (IQR)	3 (2-3)	3 (2-3)	2 (2-3)	2.5 (2-3)	3 (2-3)				
	1=mostly negative, n (%)	7 (3.3)	6 (3.1)	2 (2)	4 (7)	0 (0)				
	2=equal positive and negative effects, n (%)	98 (45.6)	88 (45.8)	47 (55)	23 (43)	18 (34)				
	3=mostly positive, n (%)	110 (51.2)	98 (51)	36 (42)	27 (50)	35 (66)				
**After the COVID-19 outbreak, would you say technology has had a mostly positive effect on our society or a mostly negative effect on our society?**								0.2518^d^		2.8 (2)
	Score, median (IQR)	3 (2-3)	3 (2-3)	3 (2-3)	3 (2-3)	3 (2-3)				
	1=mostly negative, n (%)	6 (2.8)	5 (2.6)	0 (0)	5 (9)	0 (0)				
	2=equal positive and negative effects, n (%)	78 (36.1)	69 (35.8)	33 (38)	19 (35)	17 (32)				
	3=mostly positive, n (%)	132 (61.1)	119 (61.7)	53 (62)	30 (56)	36 (68)				

^a^Responses from participants who identified as Chinese, Korean, Vietnamese, Taiwanese, and multiracial and a participant who specified Asian race and ethnicity different from those listed previously.

^b^Responses from participants who identified as Chinese, Korean, and Vietnamese.

^c^Responses from participants who identified as Chinese, Korean, or Vietnamese.

^d^Kruskal-Wallis rank sum test.

^e^Fisher exact test.

^f^N/A: not applicable.

^g^Overall, 1 missing response from the Chinese group; total sample size=215; subtotal sample size=192; Chinese sample size=85; Korean sample size=54; Vietnamese sample size=53.

^h^Total sample size=215; subtotal sample size=192; Chinese sample size=85; Korean sample size=54; Vietnamese sample size=53.

### Apps Used for Written and Audio or Video Communication

Table 2 shows the apps used for written and audio or video communication by older Asian American immigrant adults and Chinese, Korean, and Vietnamese groups. Approximately half of the participants (103/212, 48.6% older Asian American immigrants; 91/189, 48.1% across Chinese, Koreans, and Vietnamese) used email for written communication, with email use at 44% (37/85) for Chinese participants, 57% (31/54) for Korean participants, and 46% (23/50) for Vietnamese participants. Most participants used mobile phone texting for written communication (131/212, 61.8% older Asian American immigrants; 118/189, 62.4% across Chinese, Koreans, and Vietnamese; 42/85, 49% Chinese; 44/54, 82% Koreans; 32/50, 64% Vietnamese). The following results were regarding written communication apps and audio or video communication apps. Chinese participants used WeChat the most for written communication (65/85, 77%) and audio or video communication (57/84, 68%) among the apps. Korean participants were the only participants who reported having used KakaoTalk with most use for written communication (49/54, 91%) and audio or video communication (49/54, 91%). Vietnamese participants mostly reported the use of Facebook Messenger (32/50, 64%) for written communication and Apple Face Time (33/50, 66%) or Facebook Messenger (31/50, 62%) for audio or video communication. Some participants did not use any of the written communication apps (20/212, 9.4% older Asian American immigrants; 17/189, 8.9% across Chinese, Koreans, and Vietnamese); 18% (9/50) of Vietnamese participants, 7% (6/85) of Chinese participants, and 4% (2/54) of Korean participants did not use written communication apps. Some participants did not use any of the audio or video communication apps (22/211, 10.4% older Asian American immigrants; 20/188, 10.6% across Chinese, Koreans, and Vietnamese); 20% (10/50) of Vietnamese participants, 11% (9/84) of Chinese participants, and 2% (1/54) of Korean participants did not use audio or video communication apps.

**Table 2:** Apps used for written and audio or video communication by older Asian American immigrant adults and by Chinese, Korean, and Vietnamese groups.

Variables			Total older Asian American immigrants, n (%)^a^		Subtotal across older Chinese, Korean, and Vietnamese immigrants, n (%)^b^		Chinese immigrants, n (%)^c^		Korean immigrants, n (%)^c^		Vietnamese immigrants, n (%)^c^
**What communication apps are you using for written communication?^d^ (multiple responses)**											
	Email	103 (48.6)		91 (48.1)		37 (44)		31 (57)		23 (46)	
	Mobile phone texting	131 (61.8)		118 (62.4)		42 (49)		44 (82)		32 (64)	
	Facebook Messenger	49 (23.1)		44 (23.3)		7 (8)		5 (9)		32 (64)	
	WhatsApp	11 (5.2)		9 (4.8)		9 (11)		0 (0)		0 (0)	
	WeChat	74 (34.9)		66 (34.9)		65 (77)		1 (2)		0 (0)	
	KakaoTalk	49 (23.1)		49 (25.9)		0 (0)		49 (91)		0 (0)	
	Line	24 (11.3)		10 (5.3)		10 (12)		0 (0)		0 (0)	
	Specified a written communication app different from abovementioned ones (ie, Twitter, Google Chat, Skype, LinkedIn, Telegram, Zalo, Viber, Instagram, and TikTok)	29 (13.7)		27 (14.3)		6 (7)		4 (7)		17 (34)	
	None	20 (9.4)		17 (8.9)		6 (7)		2 (4)		9 (18)	
**What communication apps are you using for audio/video communication?^e^ (multiple responses)**											
	Apple FaceTime	83 (39.3)		73 (38.8)		21 (25)		19 (35)		33 (66)	
	Video Android	20 (9.5)		19 (10.1)		6 (7)		13 (24)		0 (0)	
	Facebook Messenger	39 (18.5)		38 (20.2)		3 (4)		4 (7)		31 (62)	
	Zoom	31 (14.7)		29 (15.4)		13 (16)		16 (30)		0 (0)	
	WeChat	65 (30.8)		58 (30.9)		57 (68)		1 (2)		0 (0)	
	KakaoTalk	49 (23.2)		49 (26.1)		0 (0)		49 (91)		0 (0)	
	Line	20 (9.5)		7 (3.7)		7 (8)		0 (0)		0 (0)	
	Specified an audio/video communication app different from abovementioned ones (ie, Skype, WhatsApp, Telegram, Zalo, Viber, Tango, and FCC HD)	24 (11.4)		21 (11.2)		8 (10)		2 (4)		11 (22)	
	None	22 (10.4)		20 (10.6)		9 (11)		1 (2)		10 (20)	

^a^Responses from participants who identified as Chinese, Korean, Vietnamese, Taiwanese, and multiracial and a participant who specified Asian race and ethnicity different from those listed previously.

^b^Responses from participants who identified as Chinese, Korean, and Vietnamese.

^c^Responses from participants who identified as Chinese, Korean, or Vietnamese.

^d^Overall, 4 missing responses, of which 1 (25%) was from the Chinese group and 3 (75%) were from the Vietnamese group; total sample size=212; subtotal sample size=189; Chinese sample size=85; Korean sample size=54; Vietnamese sample size=50.

^e^Overall, 5 missing responses, of which 2 (40%) were from the Chinese group and 3 (60%) were from the Vietnamese group; total sample size=211; subtotal sample size=185; Chinese sample size=84; Korean sample size=54; Vietnamese sample size=50.

### Experience With and Attitudes Toward Telehealth

Table 3 summarizes the experiences and attitudes of older Asian American immigrant adults and Chinese, Korean, and Vietnamese groups toward telehealth. Overall, approximately one-fourth of the older Asian American immigrant adults (48/215, 22.3%; across Chinese, Korean, and Vietnamese groups: 42/192, 21.9%) already had a telehealth appointment, with Korean participants at 28% (15/54), Chinese participants at 25% (21/85), and Vietnamese participants at 11% (6/53). There were significant differences among the groups (*P*=.0005) that expressed they would never consider trying a telehealth appointment. Just less than half of the older Asian American immigrant adults (95/215, 44.2%; 87/192, 45.3% across Chinese, Koreans, and Vietnamese groups; 22/85, 26% Chinese; 24/54, 44% Koreans; 41/53, 77% Vietnamese) reported that they would never consider trying a telehealth appointment. Participants were able to choose >1 response regarding specific concerns about telehealth services. More than half of the participants worried about the quality of health care (121/212, 57.1% older Asian American immigrants; 110/190, 57.9% across Chinese, Koreans, and Vietnamese; 34/83, 41% Chinese; 30/54, 56% Koreans; 46/53, 87% Vietnamese), less than half of the participants were not convinced that a telehealth diagnosis can ever be truly accurate (93/212, 43.9% older Asian American immigrants; 81/190, 42.6% across Chinese, Koreans, and Vietnamese; 27/83, 33% Chinese; 23/54, 43% Koreans; 31/53, 59% Vietnamese), and approximately one-third of the participants have never used telehealth services before and do not know how to start (68/212, 32.1% older Asian American immigrants; 61/190, 32.1% across Chinese, Koreans, and Vietnamese; 13/83, 16% Chinese; 20/54, 37% Koreans; 28/53, 53% Vietnamese). There were significant differences in perspectives regarding the main advantages of telehealth services among Chinese, Korean, and Vietnamese groups (*P*=.0005). Approximately half of the total older Asian American immigrant adults (102/214, 47.7%) reported no need for transportation as the main advantage of telehealth services, whereas approximately all Vietnamese participants (47/53, 89%) selected this reason as the main advantage, as compared with half of Korean participants (28/54, 52%), and less than one-fourth of Chinese participants (20/85, 24%). In total, less than half of the participants (95/215, 44.2% older Asian American immigrants; 81/192, 42.2% across Chinese, Koreans, and Vietnamese; 24/85, 28% Chinese; 22/54, 41% Koreans; 35/53, 66% Vietnamese) reported that a telehealth visit will never match an in-person visit. Furthermore, 39% (33/85) of Chinese participants reported that although telehealth does not compare with in-person visits, it Is a good option for initial consultation or basic care; followed by 28% (15/54) Korean participants and 17% (9/53) Vietnamese participants. There were significant differences in having the COVID-19 outbreak change the perspectives about telehealth use among groups (*χ*^2^_2_=20.6; *P*<.0001), and the median was 2 (IQR 2-3) for the Chinese group, 1 (IQR 1-3) for the Korean group, and 2 (IQR 1-2) for the Vietnamese group. Most Korean participants (33/54, 61%) reported to be less likely to use telehealth, approximately half of Chinese participants reported having the same opinion as before the COVID-19 outbreak (47/85, 55%), more than half of Vietnamese participants reported having the same opinion as before the COVID-19 outbreak (29/52, 56%), and more than one-fourth of Chinese participants (25/85, 29%) reported to be more likely to use telehealth in the future.

**Table 3:** Experience and attitudes of older Asian American immigrant adults and Chinese, Korean, and Vietnamese groups toward telehealth.

			Total older Asian American immigrants^a^		Subtotal across older Chinese, Korean, and Vietnamese immigrants^b^		Chinese immigrants^c^		Korean immigrants^c^		Vietnamese immigrants^c^		*P* value			Chi-square (*df*)^d^	
**Have you considered trying a telehealth appointment?**														.0005^e^			N/A^f^
	Sample size, n	215		192		85		54		53							
	No, and I would never consider a telehealth appointment, n (%)	95 (44.2)		87 (45.3)		22 (26)		24 (44)		41 (77)							
	No, but I would consider a telehealth appoint, n (%)	37 (17.2)		31 (16.1)		20 (24)		7 (13)		4 (8)							
	Yes, I have considered it, but I have not yet had an appointment, n (%)	35 (16.3)		32 (16.7)		22 (26)		8 (15)		2 (4)							
	Yes, and I have already had a telehealth appointment, n (%)	48 (22.3)		42 (21.9)		21 (25)		15 (28)		6 (11)							
**Does anything in particular concern you about telehealth services?^g^ (multiple responses)**														N/A			N/A
	Sample size, n	212		190		83		54		53							
	I worry about the quality of health care, n (%)	121 (57.1)		110 (57.9)		34 (41)		30 (56)		46 (87)							
	I am not convinced a telehealth diagnosis can ever be truly accurate, n (%)	93 (43.9)		81 (42.6)		27 (32.5)		23 (43)		31 (59)							
	I do not want my appointment to be recorded, n (%)	12 (5.7)		10 (5.3)		8 (10)		1 (2)		1 (2)							
	I worry about the privacy of my personal health information, n (%)	21 (9.9)		19 (10)		15 (18)		1 (2)		3 (6)							
	I do not have an electronic device to access telehealth services, n (%)	26 (12.3)		25 (13.2)		5 (6)		12 (22)		8 (15)							
	I have never used telehealth services before and do not know how to start, n (%)	68 (32.1)		61 (32.1)		13 (16)		20 (37)		28 (53)							
	A medical interpreter is not available for me, n (%)	19 (9)		14 (7.4)		12 (15)		0 (0)		2 (4)							
	Specified reason is different from abovementioned ones, n (%)	27 (12.7)		25 (13.2)		16 (19)		9 (17)		0 (0)							
**What do you view as the main advantage to telehealth services?**														.0005^c^			N/A
	Sample size, n	215		192		85		54		53							
	Quicker access to care, n (%)	52 (24.3)		46 (23.9)		32 (38)		9 (17)		5 (9)							
	Greater access to care in remote areas, n (%)	14 (6.5)		13 (6.8)		13 (15)		0 (0)		0 (0)							
	No need for transportation, n (%)	102 (47.7)		95 (49.5)		20 (24)		28 (52)		47 (89)							
	The ability to take less time out of my day, n (%)	32 (14.9)		28 (14.6)		10 (12)		17 (32)		1 (2)							
	Avoid overcrowding of waiting rooms, n (%)	14 (6.5)		10 (5.2)		10 (12)		0 (0)		0 (0)							
**Which of the following might deter you from making a future telehealth appointment?^h^ (multiple responses)**														N/A			N/A
	Sample size, n	210		188		81		54		53							
	I just prefer to meet with someone in person, n (%)	158 (75.2)		139 (73.9)		43 (53)		44 (82)		52 (98)							
	Greater access to care in remote areas, n (%)	18 (8.6)		18 (9.6)		9 (11)		9 (17)		0 (0)							
	I do not want to mess with technology, n (%)	49 (23.3)		47 (25)		18 (22)		7 (13)		22 (42)							
	I am not convinced that someone could give good health care by telehealth, n (%)	58 (27.6)		54 (28.7)		17 (21)		4 (7)		33 (62)							
	I do not think my internet connection is good enough, n (%)	19 (9)		19 (10.1)		7 (9)		7 (13)		5 (9)							
**Do you feel that people get comparable health care through telehealth as they do for in-person visits?**														.0015^c^			N/A
	Sample size, n	215		192		85		54		53							
	No, telehealth care will never match the quality of an in-person visit, n (%)	95 (44.2)		81 (42.2)		24 (28)		22 (41)		35 (66)							
	No, but telehealth is a good option for initial consultation or basis care, n (%)	60 (27.9)		57 (29.7)		33 (39)		15 (28)		9 (17)							
	Yes I think the care is comparable, n (%)	41 (19.1)		37 (19.3)		16 (19)		14 (26)		7 (13)							
	I am not sure, n (%)	19 (8.8)		17 (8.9)		12 (14)		3 (6)		2 (4)							
**Has the COVID-19 outbreak changed your view of telehealth?**														<.0001^d^			20.6 (2)
	Sample size, n	214		191		85		54		52							
	Score, median (IQR)	2 (1-2)		2 (1-2)		2 (2-3)		1 (1-3)		2 (1-2)							
	1=I am less likely to use telehealth, n (%)	72 (33.6)		67 (35.1)		13 (15)		33 (61)		21 (40)							
	2=I have the same opinion compared to before the COVID-19 outbreak, n (%)	91 (42.5)		81 (42.4)		47 (55)		5 (9)		29 (56)							
	3=I am more likely to use telehealth, n (%)	51 (23.8)		43 (22.2)		25 (29)		16 (30)		2 (4)							
**Would you wear a smartwatch to help your doctor track your symptoms between appointments?**														.0128^d^			8.7 (2)
	Sample size, n	215		192		85		54		53							
	Score, median (IQR)	2 (1-4)		2 (1-3.2)		1 (1-4)		3 (1-4)		2 (1-2)							
	1=not likely, n (%)	103 (47.9)		92 (47.9)		45 (53)		23 (43)		24 (45)							
	2=somewhat likely, n (%)	31 (14.4)		31 (16.1)		6 (7)		2 (4)		23 (43)							
	3=likely, n (%)	26 (12.1)		21 (10.9)		12 (14)		3 (6)		6 (11)							
	4=very likely, n (%)	55 (25.6)		48 (25)		22 (26)		26 (48)		0 (0)							

^a^Responses from participants who identified as Chinese, Korean, Vietnamese, Taiwanese, and multiracial and a participant who specified Asian race and ethnicity different from those listed previously.

^b^Responses from participants who identified as Chinese, Korean, and Vietnamese.

^c^Responses from participants who identified as Chinese, Korean, or Vietnamese.

^d^Kruskal-Wallis rank sum test.

^e^Fisher exact test.

^f^N/A: not applicable.

^g^Overall, 4 missing responses, of which 3 (75%) were from the Chinese group and 1 (25%) was from Asian race and ethnicity was different from those listed previously.

^h^Overall, 6 missing responses, of which 5 (83%) were from the Chinese group and 1 (17%) was from Asian race and ethnicity was different from those listed previously.

### Psychosocial Needs and Effects of the COVID-19 Pandemic

Multimedia Appendix 4 summarizes the psychosocial needs of and effects of the COVID-19 pandemic on older Asian American immigrant adults and Chinese, Korean, and Vietnamese groups. There were significant differences among Chinese, Korean, and Vietnamese groups, pertaining to overall psychosocial health, social distancing, worries, and functioning. For overall psychosocial health with regard to how well they have been able to concentrate or focus during the COVID-19 outbreak (1=not at all to 10=extremely well; *χ*^2^_2_=44.7; *P*<.0001), the median was 8 (IQR 8-9) for the Korean group, 6 (IQR 1-8) for the Vietnamese group, and 5 (IQR 4-6) for the Chinese group. With regard to how much they have been able to maintain social distance (1=not at all to 10=at all times; *χ*^2^_2_=33.6; *P*<.0001), the median was 10 (IQR 9-10) for the Korean group, 9 (IQR 9-10) for the Vietnamese group, and 8 (IQR 6-10) for the Chinese group. With regard to how stressful it has been to maintain social distancing owing to the COVID-19 outbreak (1=not at all stressful to 10=extremely stressful; *χ*^2^_2_=16.1; *P*=.0003), the median was 7 (IQR 5-9) for the Vietnamese group, 5 (IQR 2-9.8) for the Korean group, and 5 (IQR 1-7) for the Chinese group. For the following worry-related items (1=not at all worried to 10=extremely worried), such as how worried they have been about SARS-CoV-2 (*χ*^2^_2_=37.9; *P*<.0001), that they will be infected with SARS-CoV-2 (*χ*^2^_2_=75.5; *P*<.0001), that a family member will be infected with SARS-CoV-2 (*χ*^2^_2_=55.3; *P*<.0001), and that people around them will be infected with SARS-CoV-2 (*χ*^2^_2_=70.1; *P*<.0001), the median was 8 (IQR 7-9), 9 (IQR 9-9), 9 (IQR 9-9), and 9 (IQR 9-9), respectively, for the Vietnamese group; 7 (IQR 4-8), 7 (IQR 4-8), 7 (IQR 5-8), and 7 (IQR 5-8), respectively, for the Chinese group; and 5 (IQR 1-8), 2 (IQR 1-5), 4.5 (IQR 1-7.8), and 2 (IQR 1-6), respectively, for the Korean group. With regard to how worried they have been about not being able to afford or access food during the COVID-19 pandemic (*χ*^2^_2_=62.6; *P*<.0001), how worried they were about access to important resources, such as transportation or housing owing to the COVID-19 outbreak (*χ*^2^_2_=45.4; *P*<.0001), and how the COVID-19 crisis in their area created financial problems for participants or their family (*χ*^2^_2_=17.7; *P*=.0001), the median was 7 (IQR 5-8), 6 (IQR 1-8), and 2 (IQR 1-8), respectively, for the Vietnamese group; 3 (IQR 1-5), 3 (IQR 1-5), and 3 (IQR 1-5), respectively, for the Chinese group; and 1 (IQR 1-2), 1 (IQR 1-1), and 1 (IQR 1-1), respectively, for the Korean group. With regards to functioning, participants have experienced difficulties in life owing to the COVID-19 outbreak (1=experienced no difficulties to 10=experienced extreme difficulties; *χ*^2^_2_=51; *P*<.0001) and the distress they have had owing to the COVID-19 outbreak (1=not at all distressed to 10=extremely distressed; *χ*^2^_2_=22.1; *P*<.0001), the median was 6 (IQR 5-7) and 7 (IQR 5-7), respectively, for the Vietnamese group; 5 (IQR 2-6) and 5 (IQR 2-6), respectively, for the Chinese group; and 1 (IQR 1-3) and 4.5 (IQR 1-6.8), respectively, for the Korean group.

Most participants reported that 2 people lived in their house including themselves (112/215, 52.1% older Asian American immigrants; 99/192, 51.6% across Chinese, Koreans, and Vietnamese; 51/85, 60% Chinese; 27/54, 50% Koreans; 21/53, 40% Vietnamese). There were *significant* differences with regard to having had a family/household member’s salary, *hours*, or contracts significantly reduced (*P*=.0005) and having had a family/household member or friend fallen physically ill (*P*=.0005) owing to the COVID-19 outbreak. Most Vietnamese participants (15/53, 28%) had a family/household member’s salary, *hours*, or contracts significantly reduced, followed by Korean (8/53, 15%) and Chinese (2/81, 2%) participants. Most Korean participants (10/53, 19%) reported having had a family/household member or friend fallen physically ill, followed by Chinese (7/81, 9%) and Vietnamese (0/53, 0%) participants. There were *significant* differences among Chinese, Korean, and Vietnamese groups in how relationships have been between members of family/household during the COVID-19 outbreak (1=extremely negative to 10=extremely positive; *χ*^2^_2_=33.2; *P*<.0001), and the median was 9 (7.2-10) for the Korean group, 9 (IQR 7.8-9.2) for the Vietnamese group, and 6 (IQR 5-8) for the Chinese group. There were *significant* differences among Chinese, Korean, and Vietnamese groups regarding what the exercise activity level has been (*χ*^2^_2_=20.2; *P*<.0001) and how much they have engaged in hobbies (*χ*^2^_2_=26.6; *P*<.0001) since the COVID-19 outbreak—the median was 5 (IQR 4-5) and 6 (IQR 5-7), respectively, for the Vietnamese group; 5 (IQR 3-5) and 5 (IQR 5-5), respectively, for the Korean group; and 4 (IQR 3-5) and 5 (IQR 5-5), respectively, for the Chinese group.

## Discussion

### Principal Findings

In a group of 216 older Asian American immigrant adult participants, we found significant differences in technology access, telehealth use, and psychosocial health impacts among the Chinese, Korean, and Vietnamese groups. In our CBPR cross-sectional survey study, we examined the readiness for a web-based senior center among older Asian American immigrant adults and specifically among Chinese, Korean, and Vietnamese groups. We also identified the psychosocial needs and effects of the COVID-19 pandemic that a web-based senior center could be positioned to meet.

Socioeconomic status is an important context when planning a web-based senior center because financial resources are often limited. It is important to avoid adding financial burden while trying to be intentional in providing web-based care and social services via mobile apps. In our study, most Chinese (38/86, 44%), Korean (39/54, 72%), and Vietnamese (25/53, 47%) older participants had an unlimited mobile data plan, followed by a small group that had a limited mobile data plan. Of those using limited plans, many (39/216, 18.1%) were unsure about their data limits. More than half of older Asian American immigrant adult participants (123/216, 56.9%), including older Chinese, Korean, and Vietnamese participants, reported <US $15,000 as total household income before taxes. According to an AHSC leader, staff has assisted many older Asian American immigrant clients and applied for an affordable internet plan during the COVID-19 pandemic.

Overall, more than half of the older Asian American immigrant adult participants (131/212, 61.8%), including older Chinese, Korean, and Vietnamese participants, reported using mobile phone texting for written communication, followed by approximately half of the participants (103/212, 48.6%) using email for written communication in our study. Both mobile phone texting and email can present challenges for older adults. Challenging intrinsic factors can affect the adoption of a web-based senior center. For example, having less dexterity, experiencing tremors or physical changes resulting from arthritis or stroke, not having confidence in using new apps or platforms, and not being interested in learning new ways to access social and health services are known barriers to using technology for health purposes [30-32]. Extrinsic barriers include limited to no access to digital communication devices for all older adults, not having trust in technology, belief that mobile phones are for communication rather than accessing health and social services, and cultural beliefs that technologies detract from family time [31] or may dismantle cultural expectations for children and grandchildren to care for their older family members as they age [17].

There is a gamut of available information and communication technology devices and apps, including written and audio or video. We found that only 2% (1/53) of the Vietnamese older participants started using a new electronic device to communicate with friends and family after the COVID-19 outbreak, followed by more than half of Korean older participants (31/54, 57%) and less than one-fifth of Chinese older participants (15/86, 17%). This may be owing to not knowing what is available, not knowing how to use the device, or the degree of comfort with use. Our findings differ from those of a study that focused on South Koreans, where researchers identified themes that included a reluctance to learn about and use new technology and ambivalence regarding using technology-enabled services for connection with family or acquaintances [10]. In our study, for written and audio or video communication apps, most older Chinese participants (65/85, 77%; 57/84, 68%, respectively) used WeChat, most Korean participants (49/54, 91%; 49/54, 91%, respectively) used KakaoTalk (this app was exclusively used by the Korean group), and most Vietnamese participants used Facebook Messenger for written communication (32/50, 64%) and Apple Face Time or Facebook Messenger for audio or video communication (33/50, 66%; 31/50, 62%, respectively). Our results suggest that there are differences among groups that must be considered by CBOs offering web-based care and social services. Different communities tend to use specific communication platforms and have preferences regarding the types of services that are acceptable when using web-based platforms. It is important to note that, early in the COVID-19 pandemic, there was no existing cross-cultural communication system that was free, quickly available, and easy for symptom monitoring of large, diverse populations [33]. For example, in China, WeChat is mostly well known and is among the frequently used, web-based, health service social media platforms [34]. Our community-academic partnership kept in mind that individual COVID-19 prevention and control apps, such as WeChat in China, were developed by adding to existing social apps with regard to the management of the COVID-19 outbreak [35]. KakaoTalk is a mobile instant messenger based in South Korea (ie, host country) and is the most popular and cross-platform social media service in South Korea [36]. In our study, findings imply that cultural and country-based web-based communication platforms, such as WeChat and Kakao Talk, are important sustainable connections with diverse Asian immigrant groups [34-36] for sustainable accessibility [37]. Our study results suggest that culturally based CBOs serving diverse communities need to navigate community contexts, capacity, and operations and determine the capacity for providing sustainable cultural, linguistic, and health care services via web-based care. CBOs and researchers need to consider how to best use these platforms, given that personal health information may be a part of certain communications. Authenticity and intentionality will be needed regarding which web-based services are best suited for these various platforms.

Web-based care is different from in-person care. In our study, few older Asian American participants, including Chinese, Korean, and Vietnamese participants perceived themselves to be very technology savvy. Furthermore, most Vietnamese older participants (41/53, 77%) expressed that they would never consider trying a telehealth appointment. A few older Chinese and Korean participants expressed the same view. Thus, CBOs and researchers need to consider intentionally using multiple communication platforms; ones that each community group is already familiar with. This will likely improve sustainability because it will relieve community members from having to learn something new to access health and social services.

We made additional important contributions to the literature about what to consider regarding sustaining accessibility in telehealth. We found that more than half of older Asian American immigrant adults (121/212, 57.1%) worry about the quality of health care with web-based care and social services. Less than half (93/212, 43.9%) were not convinced that a diagnosis made via telehealth would result in an accurate diagnosis. For example, most Vietnamese among Asian American immigrants (28/53, 53%) had never used telehealth services and do not know how to start. These findings align with what CBOs have reported—that is, most older Asian American clients struggled to use web-based platforms and web-based programs and had limited technological literacy despite having compatible computers and platforms [6]. Of importance, according to older Chinese, Korean, and Vietnamese immigrants, the main advantage of telehealth was not needing transportation services. Implications from our study suggest that there is a need to further enhance older Asian American immigrant clients’ readiness for a web-based senior center, and one way is to engage and collaborate with more clients in subsequent intervention design and training in technology and telehealth delivery. According to AHSC community partners, regarding their work with older Asian American immigrant adult clients, they expressed the importance to build trust over time. For example, there are clients who were more willing to share concerns after their staff built personal connections.

Regarding overall psychosocial health in our study, older Chinese immigrants had a reduced ability to concentrate or focus during the COVID-19 outbreak. Furthermore, older Chinese, Koreans, and Vietnamese engaged less in exercise and hobbies. According to a program manager at AHSC, many older Chinese clients enjoyed being at the center in person for physical activity (eg, Tai Chi and light aerobics) and hobbies (eg, singing, dancing, and social groups) before the outbreak. A few older Chinese participants expressed that they did not have a place to go to for physical activity or engaging in hobbies. Some chose to stay at home and away from crowds owing to infection precautions, and in particular, Chinese older adults wanted to avoid anti-Asian hate. AHSC community leaders examined anti-Asian hate more specifically in another initiative apart from what our community-academic partnership’s CBPR research cross-sectional survey study aimed in this study. Our partnership remains cognizant that among the anti-Asian hate incidents was the use of the terms, China virus or Wuhan virus, which relates a virus with a race, ethnicity, or city instead of to the biological SARS-CoV-2 or COVID-19, and this does not align with the World Health Organization [37]. Researchers discovered profound discrimination and violence among Asian American populations; for example, Chinese and Vietnamese commonly experienced being yelled at and being given *dirty looks* for carrying the virus [3]. Our findings suggest that there may be a need for increased caregiving efforts; a need for caregiver support; and a need for increasing services for social, health, and financial stressors; however, the extent is different among groups. For example, most Vietnamese immigrants, followed by Chinese and Korean immigrants, experienced stress owing to maintaining social distancing, worry about SARS-CoV-2, and worry about infecting themselves and people. In another example, most Vietnamese immigrants, followed by Chinese and Korean immigrants, worry about not being able to afford or access food and important resources, such as transportation or housing; feel that the crisis created financial problems for them or their family; experienced difficulties in life and distress; and had a family/household member’s salary, hours, or contracts significantly reduced. These results align with the findings by Quach et al [3] and Tiwari and Zhang [7]. CBOs should include psychosocial services in the web-based portfolio. Psychosocial services will likely need to be administered on different platforms to different community groups. Psychosocial services will need to be tailored to the specific needs of each community group because they would be different across groups.

To meet these needs and to support safety while offering the broadest possible access to care, CBOs may wish to consider rebuilding after the pandemic by adding web-based health and social services for older adults. To reduce barriers for clients, consideration should be given to the needs of specific cultural groups and the technology platforms already in use by each group. A CBO web-based senior center should be designed for use across multiple free platforms such as Facebook, WeChat, KakaoTalk, or any other platform used by a specific group that a CBO serves. An important component of intentional planning and design includes conducting a survey to discover which social media platforms the CBOs clientele is familiar with and which ones they trust. Delivering services across multiple platforms may add burden to CBOs, but it will improve access and acceptance of web-based programming, thus providing the opportunity to extend reach and support more older adults and their families.

### Limitations and Future Studies

Although the timing of the survey limits this study in part owing to recall consideration, this is an important step. We conducted the survey in the second year of the COVID-19 pandemic, in 2022 [38]. Community-academic partners originally planned to implement the study starting in July of 2021, but this was not possible owing to concurrent Asian American immigrant community needs driven by the COVID-19 pandemic. We honored the need to pivot, so that AHSC could focus on addressing staffing, vaccinations, and other needs in response to the virus and the increasing anti-Asian hate. AHSC community leaders expressed that the ability to recall psychosocial impacts based on ratings from 1 to 10 may have influenced the ability for some older adult participants to differentiate between 2 numbers that are next to one another (eg, 5 vs 6). Often, in health technology studies focused on individuals of Asian descent, the research data of subgroups within the large Asian population are aggregated. We examined our study data as a large group of older Asian American immigrant adults and among Chinese, Korean, and Vietnamese participants. However, further studies are needed to examine at a large scale and longitudinally and to examine additional Asian subgroups, for example, Taiwanese and multiracial groups, as they may have different needs. The response rate and completion rate were high in our study, even though a small portion of clients declined to participate. Reasons for rejection included survey length and not having experience with technology or telehealth, and, according to AHSC leaders, some may have declined because of not having a need to use a technology to access health care or having had a bad experience with technology. Although the instruments that we adapted from the PhenX Toolkit [19] have not been formally tested and validated, we pretested them with community partners for face validity and technical functionality before use in this study. We recommend further studies for additional psychometric testing and continuing engagement of older Asian American immigrants in co-designing for adoption research and building upon a CBPR approach using both quantitative and qualitative methods. This may increase meaningful use and sustainability [9,13,39]. Further studies need to address continuing engagement of older immigrant clients in building and sustaining a senior center, completely web-based versus hybrid—combination of web-based and in-person services, and essential trust in web-based continuity of health care and social service. We also recommend further examination of technology accessibility, technology literacy, and complexity of interventions as barriers to or facilitators of uptake [40] and the ethics and utility of using different types of technologies in service and clinical care from the perceptions and experiences of older Asian American immigrant adults, CBO leaders, and health care providers.

### Conclusions

Results from our community-academic partnership study inform the rebuilding of an efficacious web-based senior center, where more older Asian American immigrant adults who need can obtain access to the internet and education about using technology-enabled communication devices. Differences in psychosocial needs and the effects of the COVID-19 pandemic were reported among Chinese, Korean, and Vietnamese groups. The strength of the participating older adults was observed and honored. There is a need to engage clients and culturally diverse CBOs in technology access and telehealth as a part of bridging care. This includes uplifting the communication about clients’ health and extending the reach of providing care remotely through distance learning and distance integrative health care and social service delivery. There are different psychosocial needs and effects of the COVID-19 pandemic that a web-based senior center could be positioned to meet. Consideration should be given to intragroup and intergroup needs across older Asian American immigrant adults such as among Chinese, Korean, and Vietnamese groups within the large older group. Our study results illuminate the conventional challenges in delivering health care since the COVID-19 pandemic and a pathway forward for improving care and advancing health equity for culturally diverse, older, Asian immigrants.

## Appendix

Multimedia Appendix 1 Combined consent; eligibility; and technology access, telehealth, and psychosocial health impacts survey—Microsoft Word document version.

Multimedia Appendix 2 Combined consent; eligibility; and technology access, telehealth, and psychosocial health impacts survey—PDF version.

Multimedia Appendix 3 Sociodemographics and background characteristics of older Asian American immigrant adults and Chinese, Korean, and Vietnamese groups.

Multimedia Appendix 4 Psychosocial needs of and effects of the COVID-19 pandemic on older Asian American immigrant adults and Chinese, Korean, and Vietnamese groups.

## Abbreviations

AHSC: Asian Health & Service Center
CBO: community-based organization
CBPR: community-based participatory research
CHW: community health worker
PI: principal investigator
WSU: Washington State University
