# Supplementary material for: Toward Sustaining Web-Based Senior Center Programming Accessibility With and for Older Adult Immigrants: Community-Based Participatory Research Cross-Sectional Study
Source: Asian Pac Isl Nurs J. 2024 Jan 26;8:e49493. doi: 10.2196/49493 (PMC10858412; doi:10.2196/49493)
Supplement: Multimedia Appendix 2 [file apinj_v8i1e49493_app2.pdf]

WASHINGTON STATE UNIVERSITY
College of Nursing

Asian Health & Service Center
華人服務中心
아시안 보건 복지 센터
Trung Tâm Y Tế & Dịch Vụ Châu Á

Note to Research Team Partnership for the Qualtrics Online Survey: 20 screens total, of which 17 screens for the survey for Parts A – Sociodemographic and Background, B – Technology Accessibility, C – Technology Telehealth Use, and Part D – Psychosocial Health. The phrase, "Need to click here to go to the next page", is at the end of the research study information consent and eligibility screen. The following instructional phrases were included on each screen for the survey for Parts A through D: "Need to click here to go the previous page" and "Need to click here to go to the next page". The phrase, "Need to click here to go to the next page", is at the end of the review step and contact information screen.

Research Study Information Consent

Research Study Title: Building an Effective Virtual Senior Center Through Technology Solutions: Accessibility, Telehealth Utility, and Psychosocial Health [Bridging through Cultural Telehealth]: COVID-19 Pandemic and Beyond

This research study has been certified as exempt from the need for review by the Washington State University (WSU) Institutional Review Board.

Purpose of the research study: Principal Investigators Connie Kim Yen Nguyen-Truong, PhD, RN, ANEF, and Roschelle L. Fritz, PhD, RN, FAAN, at WSU College of Nursing in partnership with Holden Leung, MSW, at Asian Health & Service Center are conducting a community-based participatory research (CBPR) study. We are learning what kinds of technology you have and are able to access (technology accessibility); what kinds of technology that you use in general for health before and after the COVID-19 outbreak (telehealth utility); and your thoughts on social distancing, hopes and worries, families and relationships, daily activities, health concerns, and functioning, and the psychosocial health impact during the COVID-19 pandemic (psychosocial health) by diverse older Asian immigrant adults ages 60 years and older in light of the COVID-19 pandemic. Findings from this research study will help us to understand the best ways for developing a virtual senior center and addressing community needs and strengths. About 250 older Asian immigrant adults will participate in this online research survey study. We will develop solutions for building an effective virtual senior center.

Eligibility to participate: You are eligible to participate if you are ages 60 years or older.

Funders: Washington State University Vancouver including the Research Mini-Grant

Your participation in the research study is completely voluntary. There will be no penalty or loss of services or benefits if you decide to not take part in the study or quit later.

What you will be doing in this research study:

- You will complete an online survey that will take about 30 minutes to complete but may take shorter or longer time.

- You can choose to have interpretation support from Asian Health & Service Center. We can assist you as you complete the online survey by telephone or video call such as zoom. The survey will not be done in-person.
    - You can choose whether to turn on your camera to show your face or image during a video call. There will be no recording.
- There are 4 parts to this survey:
    - Part A – Sociodemographic and Background: These questions are about you so we can better describe the group that participated in this research study.
    - Part B – Technology Accessibility: These questions are about what kinds of technology you have and are able to access.
    - Part C – Technology Telehealth Use: These questions are about what kinds of technology that you use in general for health before and after the COVID-19 outbreak.
    - and Part D – Psychosocial Health: These questions are about your thoughts on social distancing, hopes and worries, families and relationships, daily activities, health concerns, and functioning, and the psychosocial health impact during the COVID-19 pandemic.
- If you do not know the answer to a question, please give your best estimate.
- You can skip any question that you are uncomfortable answering.
- All of your responses will be kept confidential. Questions that are answered by you will be saved, and you will see a submission confirmation message.

There will be no cost to you for taking part in this research online survey study. You will receive a $10 shopping gift card (for example, Fred Meyer) upon completion of the research study to honor and thank you for your time. Asian Health & Service Center Collaborator will talk with you regarding the gift card upon completion. If you decide to quit the research study, you will receive no further payment.

<u>The potential benefits to you for participating in this research study:</u> If you take part in this research study, you may learn about advocating for yourself and may help others in the future. There is no direct or intended benefit to you from being in this research study. Thank you for your valuable time.

<u>Research risks and privacy:</u>

- The potential risks from taking part in this research study are emotional discomfort or a feeling of embarrassment from providing responses in the survey.
- There is the potential of loss of privacy if you choose to have interpretation support from Asian Health & Service Center assist you.
- No actual names will be obtained with the survey. Participant identification numbers will be used and a key maintained separately to follow-up and provide participant compensation.
- The data for this research study will be kept confidential to the extent allowed by federal and state law.
- In the event of any publication or presentation resulting from the research, no personally identifiable information will be shared because your name is not linked to your responses.
- Your survey responses are entered into a secure online database called Qualtrics at WSU.
- The information we collect from each participant is stored with a unique ID number on WSU's secure server. Research study data will be kept in the research study folder on the secure server with a firewall and password-protected WSU SharePoint cloud and approved Box that is HIPAA compliant.
- Access to the file containing your contact information and link between your name and research study number is only granted to researchers and the file will be stored in the research study folder on the

secure server with a firewall and password-protected WSU SharePoint cloud or approved Box that is HIPPA compliant.
- The data for this research study will be kept for 3 years.
- The following people will have access to the data, including: all researchers, Washington State University, Asian Health & Service Center, and Institutional Review Board.

For more information or questions, please contact: Asian Health & Service Center Collaborator Holden Leung, [email], [phone]. You can also contact Principal Investigators Connie Nguyen-Truong, PhD, RN, ANEF, [email], [phone], or Roschelle Fritz, PhD, RN, FAAN, [email], [phone], at WSU. If you have questions about your rights as a research participant, or would like to report a concern or complaint about this research study, you can contact WSU Institutional Review Board at (509) 335-7646 or e-mail irb@wsu.edu.

By selecting "yes", I confirm that I understand the information in this form, I am 60 years or older, and consent to take part in the online survey research study.

I consent to take part in this research study.

☐$_1$ No → If you chose to decline to participate in this research study, please share your reason. [online] Please click on the next page button below to proceed with submitting your reason or to exit this form.

____

Thank you for your time. We respect your decision to decline to participate in this research study. [online] Please click on the next page button to submit your reason for not participating in this research study or if you are exiting this form.

☐$_2$ Yes → If you chose to participate in research study, then [online] please click on the next page button below to proceed with completing the Survey following this research study information consent form.

Research Study Title: Building an Effective Virtual Senior Center Through Technology Solutions: Accessibility, Telehealth Utility, and Psychosocial Health [Bridging through Cultural Telehealth]: COVID-19 Pandemic and Beyond

You will be taking an online survey. A team member will assist to enter the survey online through Washington State University Qualtrics.

Before you begin the survey, the following information is needed for your participation in the research study.

Please enter today's date in numbers using the format month/day/year (mm/dd/yyyy). ___________

What is the first and last name of the team member who is assisting you with the survey? ___________________

What is your participant research study number? This number is assigned to identify your answers without using any of your personal information. ☐☐☐

Part A: Sociodemographic and Background

The following are 18 questions about you so we can better describe the group that participated in this research study. It will take about 5 minutes to complete.

(1) What is your race/ethnicity? Please select all options that apply to you.

☐1 Chinese

☐2 Korean

☐3 Vietnamese

☐77 Please specify additional race/ethnicity: _______________________________________

(2) What is your age in years? ☐☐☐

(3) Where were you born?

☐1 China

☐2 Korea

☐3 Vietnam

☐77 If not listed above, please specify: _______________________________

☐99 Not sure

(4) At what age (years) did you move to the United States to live? ☐☐☐

(5) Which State in the United States do you currently live in?

☐1 Oregon

☐2 Washington

☐77 If not listed above, please specify: ________________________________

(6) How many years have you lived in the United States? (actual count of the number of years) ☐☐

(7) What is your gender?

☐1 Woman

☐2 Man

☐3 Non-binary

☐4 Prefer not to share

☐77 If not listed above, please specify: _______________________________

(8) What is your relationship status?

☐1 Single, have never been married

☐2 Married

☐3 Not married, living with a partner

☐4 Separated

☐5 Divorced

☐6 Widowed

(9) What is your highest level of education?

☐1 No school

☐2 Some elementary

☐3 Completed elementary

☐4 Some middle school

☐5 Completed middle school

☐6 Some high school

☐7 Graduated from high school

☐8 Some college

☐9 Graduated from college/Professional degree

☐10 Some graduate school

☐11 Graduated from graduate school/Professional degree

(10) What is your current employment?

☐1 Not employed

☐2 Retired

☐3 Part-time

☐4 Full-time

(10a) What is or was your occupation/type of work? The following are main occupation/type of work categories. Please select all options that apply to you.
You can go to the U.S. Bureau of Labor for sub-categories if you need to review what is within the main categories (https://www.bls.gov/emp/tables/emp-by-detailed-occupation.htm).

☐1 Management occupations

☐2 Business and financial operations occupations

☐3 Computer and mathematical occupation

☐4 Architecture and engineering occupations

☐5 Life, physical, and social science occupations

☐6 Community and social service occupations

☐7 Legal occupations

☐8 Educational instruction and library occupations

☐9 Arts, design, entertainment, sports, and media occupations

☐10 Healthcare practitioners and technical occupations

☐11 Healthcare support occupations

☐14 Building and grounds cleaning and maintenance occupations

☐15 Personal care and service occupations

☐16 Sales and related occupations

☐17 Office and administrative support occupations

☐18 Farming, fishing, and forestry occupations

☐19 Construction and extraction occupations

☐20 Installation, maintenance, and repair occupations

☐21 Production occupations

☐22 Transportation and material moving occupations

☐23 Household partner

☐24 Household parent

☐12 Protective service occupations

☐13 Food preparation and serving related occupations

☐25 No working history

(11) What is your total household income before taxes?

☐1 Less than $15,000

☐2 $15,000-30,000

☐3 $30,001-50,000

☐4 $50,001-60,000

☐5 $60,001-75,000

☐6 $75,001-100,000

☐7 $100,001-150,000

☐8 Equal or more than $150,001

☐99 Not Sure

(12) How well do you speak English?

☐1 Not at all

☐2 Not well

☐3 Well

☐4 Very well

(13) What language do you prefer when speaking with your healthcare provider?

☐1 Chinese Cantonese

☐2 Chinese Mandarin

☐3 Korean

☐4 Vietnamese

☐5 English

☐77 If not listed above, please specify: ____________________________

(14) How well do you write in your preferred language?

☐1 Not at all

☐2 Not well

☐3 Well

☐4 Very well

(15) Do you currently have health insurance?

☐1 No → If you chose a No response, then Go to Question 17

☐2 Yes

☐99 Not sure

(16) What kind of health insurance do you have? Please select all options that apply to you. [If you answered not having health insurance to Question 15, then skip Question 16 by not entering a response.]

❑1 Private health insurance (Employer based health insurance)

☐2 Health insurance for emergency medical care only

☐3 Medicaid/Oregon Health Plan (OHP, OHP+)

☐4 Medicare (Medicare A, B)

☐5 Other government program (Veterans Affairs, Aging and Disability Services)

☐77 Please specify additional health insurance:

____

☐99 Not sure

(17) Do you have a regular place of care for non-emergency health care services?

☐1 No → If you chose a No response, then Go to Question 19

☐2 Yes

☐99 Not sure

(18) Where do you go to seek regular, non-emergency health care services? Please select all options that apply to you. [If you answered not having a regular place of care for non-emergency health care services to Question 17, then skip Question 18 by not entering responses.]

☐1 Private Primary Care Provider office (independent practice or group practice)

☐2 Medical home

☐3 Community health service center

☐4 County health clinic

☐5 Free health clinic

☐77 Please specify additional regular, non-emergency health care services: ______________________________

☐99 Not sure

Part B: Technology Accessibility

The following 9 items are about technology accessibility. We are asking these questions because COVID-19 has presented new challenges for us in helping you to continue your visits with us. These challenges have led us to ideas on what to do now and maybe even in the future. It will take about 5 minutes to complete.

(19) How would you prefer to conduct a visit with Asian Health & Service Center? Please select your top choice.

☐1 In person

☐2 Telephone call

☐3 Video call

☐4 No preference

☐5 Decline to answer

☐77 If not listed above, please specify: ______________________________

(20) How do you currently access the internet? Please select all options that apply to you.

☐1 Smartphone (Smartphones are devices that have larger touch screens and "apps" - some examples are iPhones, Samsung Galaxy)

☐2 Tablet/ ipad

☐3 Laptop

☐4 Desktop computer

☐5 Do not currently access the internet

☐77 Please specify other access to the internet: ______________________________

(21) Do you use email to receive and send documents?

☐1 No

☐2 Yes

☐3 Decline to answer

(22) Are you interested in using any of the following electronic devices to do some parts of your visit at home? Please select all options that apply to you.

☐1 Smartphone

☐2 Tablet/ ipad

☐3 Laptop

☐4 Desktop computer

☐5 Wearable devices (for example, FitBit, Smartwatch)

☐6 Smart home devices

☐7 Not interested in using any of the above listed to do some parts of the visit at home

☐77 Please specify other technology use interests: ______________________________

(22a) Do you have any of the following electronic devices? Please select all options that apply to you.

☐1 Smartphone

☐2 Tablet/ ipad

☐3 Laptop

☐4 Desktop computer

☐5 Wearable devices (for example, FitBit, Smartwatch)

☐6 Smart home devices

☐7 No

☐99 Not sure

(23) Who is your internet provider? Please select all options that apply to you.

☐1 Century Link

☐2 Xfinity (Comcast is the parent company, and Xfinity is Comcast's brand including internet service.)

☐3 Ziply

☐4 Use a mobile phone for the internet

☐5 Go to a location that has a wifi spot (for example, coffee shop, restaurant, library)

☐6 Do not have an internet provider

☐77 Please specify other internet providers: ______________________________

(24) What communication Apps are you using for written communication? Please select all options that apply to you.

☐1 Email

☐2 Phone texting

☐3 Facebook Messenger

☐4 Twitter

☐5 Google Chat

☐6 Skype

☐7 LinkedIn

☐8 Slack

☐9 WhatsApp

☐10 WeChat

☐11 Telegram

☐12 KakaoTalk

☐13 MessengerApp Landscape China

☐14 Zalo

☐15 Line

☐16 Do not use communication Apps for written communication

☐77 If not listed above, please specify: ______________________________

(25) What communication Apps are you using for audio/video communication? Please select options that applies to you.

☐1 FaceTime – iPhone

☐2 Video - Android

☐3 Facebook Messenger

☐4 Google VideoChat

☐5 Skype

☐6 Zoom

☐7 WhatsApp

☐8 WeChat

☐9 Telegram

☐10 KakaoTalk

☐11 MessengerApp Landscape China

☐12 Zalo

☐13 Line

☐14 Do not use communication Apps for audio/video communication

☐77 If not listed above, please specify: ______________________________

(26) What type of mobile phone do you use? Please select options that applies to you.

☐1 Android (for example, Samsung Galaxy, LG, Google Pixel, or others)

☐2 iPhone

☐3 Google phone

☐4 Flip phone

☐5 Do not have a mobile phone → If you responded No, then Go to Question 28

(27) Do you have data or internet services on your mobile phone? [If you answered do not have a mobile phone to Question 26, then skip Question 27 by not entering responses.]

☐1 No

☐2 Yes

(27a) If you responded yes to Question 27 about having data or internet services on your mobile phone, please specify what data plan you have.

☐1 I have a capped/limited data plan including internet services on my mobile phone.
Please specify the amount of data you have in numbers: ____________________________

☐2 I have a capped/limited data plan including internet services on my mobile phone, but I am unsure about the amount of data I have.

☐3 I have an unlimited data plan including internet services on my mobile phone.

☐4 I am unsure if I have a capped/limited data plan or unlimited data plan.

Part C: Technology Telehealth Use

The following 23 items are about your use of technology and telehealth **Before** and **After** the COVID-19 outbreak. It will take about 10 minutes to complete.

The following items are about your use of general technology BEFORE and AFTER the COVID-19 outbreak.

**(28)** In a typical week BEFORE the COVID-19 outbreak, did you use the internet or email, at least occasionally?	☐1 No<br>☐2 Yes<br>☐3 Not sure
**(29)** In a typical week BEFORE the COVID-19 outbreak, did you have a subscription to an internet service provider at home. This could be high speed broadband service such as DSL, cable, or fiber optic service?	☐1 No<br>☐2 Yes<br>☐3 Not sure
**(30)** In a typical week BEFORE the COVID-19 outbreak, did you use a smartphone? Smartphones are devices that have larger touch screens and "apps". Some examples are iPhones, Samsung Galaxy.	☐1 No<br>☐2 Yes<br>☐3 Not sure
**(31)** In a typical week BEFORE the COVID-19 outbreak, did you use video chat like FaceTime, Skype or Zoom on your smartphone?	☐1 No<br>☐2 Yes<br>☐3 Not sure
**(32)** Do you ever use social media sites like Facebook, Twitter, or LinkedIn?	☐1 No<br>☐2 Yes<br>☐3 Not sure
**(33)** In a typical week BEFORE the COVID-19 outbreak, did you ever use messaging apps like WhatsApp, Facebook Messenger, WeChat, Snapchat?	☐1 No<br>☐2 Yes<br>☐3 Not sure

In a typical week <u>BEFORE</u> the COVID-19 outbreak, please tell us if you use any of the following items, or not. Did you use:

**(34)** A tablet like an iPad, Samsung Galaxy Tab, Microsoft Surface Pro, or Amazon Fire	☐1 No<br>☐2 Yes<br>☐3 Not sure
**(35)** A desktop or laptop computer	☐1 No<br>☐2 Yes<br>☐3 Not sure

**(36)** An activity monitor or tracker (for example, Fitbit)	☐1 No<br>☐2 Yes<br>☐3 Not sure
**(37)** A smartwatch (for example, Apple Watch or Samsung galaxy watch)	☐1 No<br>☐2 Yes<br>☐3 Not sure
**(38)** Do you consider yourself to be tech (technology) savvy?	☐1 Not at all<br>☐2 A little<br>☐3 Somewhat so<br>☐4 Very much so
**(39)** Overall, how confident do you feel using computers, smartphones, or other electronic devices to do the things you need to do online	☐1 Not at all confident<br>☐2 Only a little confident<br>☐3 Somewhat confident<br>☐4 Very confident
**(40)** Have you started using a new electronic device to communicate with friends and family AFTER the COVID-19 outbreak?	☐1 No<br>☐2 Yes
**(41)** How likely are you to participate in a research study asking you to wear a smartwatch, like an apple watch, to track your health symptoms for one year?	☐1 Not likely<br>☐2 Somewhat likely<br>☐3 Likely<br>☐4 Very likely
**(42)** BEFORE the COVID-19 outbreak, would you say technology has had a mostly positive effect on our society or a mostly negative effect on our society?	☐1 Mostly negative<br>☐2 Equal positive and negative effects<br>☐3 Mostly positive
**(43)** AFTER the COVID-19 outbreak, would you say technology has had a mostly positive effect on our society or a mostly negative effect on our society?	☐1 Mostly negative<br>☐2 Equal positive and negative effects<br>☐3 Mostly positive

The next items are about your use of telehealth BEFORE and AFTER the COVID-19 outbreak. Telehealth is getting your health care remotely by means of electronic device (smartphone, computer, iPad, or by telephone). It is also known as long-distance health care.

**(44)** Have you used telehealth services?	☐1 No<br>☐2 Yes
**(45)** Have you considered trying a telehealth appointment?	☐1 No, and I would never consider a telehealth appointment<br>☐2 No, but I would consider a telehealth appointment<br>☐3 Yes, I have considered it, but I have not yet had an appointment<br>☐4 Yes, and I have already had a telehealth appointment
**(46)** Does anything in particular concern you about telehealth services? Please select all options that apply to you.	☐1 I worry about the quality of health care<br>☐2 I am not convinced a telehealth diagnosis can ever be truly accurate<br>☐3 I do not want my appointment to be recorded<br>☐4 I worry about the privacy of my personal health information<br>☐5 I do not have an electronic device to access telehealth services.<br>☐6 I have never used telehealth services before and do not know how to start.<br>☐7 A medical interpreter is not available for me.<br>☐8 If not listed above, please specify: ______________________
**(47)** What do you view as the main advantage to telehealth services?	☐1 Quicker access to care<br>☐2 Greater access to care in remote areas<br>☐3 No need for transportation<br>☐4 The ability to take less time out of my day<br>☐5 Avoid overcrowding of waiting rooms

**(48)** Which of the following might deter you from making a future telehealth appointment (you can choose more than one)?	☐1 I just prefer to meet with someone in person<br>☐2 Greater access to care in remote areas<br>☐3 I do not want to mess with technology<br>☐4 I am not convinced that someone could give good healthcare by telehealth<br>☐5 I do not think my internet connection is good enough
**(49)** Do you feel that people get comparable health care through telehealth as they do for in-person visits?	☐1 No, telehealth care will never match the quality of an in-person visit<br>☐2 No, but telehealth is a good option for the initial consultation and/or basis care<br>☐3 Yes, I think the care is comparable<br>☐4 I am not sure
**(50)** Has the COVID-19 outbreak changed your view of telehealth?	☐1 I am less likely to use telehealth<br>☐2 I have the same opinion compared to before the COVID-19 outbreak<br>☐3 I am more likely to use telehealth
**(51)** Would you wear a smartwatch to help your doctor track your symptoms between appointments?	☐1 Not likely<br>☐2 Somewhat likely<br>☐3 Likely<br>☐4 Very likely

Part D: Psychosocial Health

The following are 37 Psychosocial Health Impact items including social distancing, hopes and worries, families and relationships, daily activities, health concerns, and functioning, and the psychosocial health impact during the COVID-19 outbreak. It will take about 10 minutes to complete.

Please answer the questions below by selecting the number that best represents how much you agree with the following statements during the COVID-19 outbreak…

(52) Has your ability to enjoy things decreased due to the COVID-19 outbreak?

Not at all decreased… …Decreased an extreme amount

1 2 3 4 5 6 7 8 9 10

(53) How well have you been able to concentrate or focus during the COVID-19 outbreak?

Not at all… …Extremely well

1 2 3 4 5 6 7 8 9 10

(54) Has your ability to solve problems decreased due to the COVID-19 outbreak?

Not at all decreased… …Decreased an extreme amount

1 2 3 4 5 6 7 8 9 10

SOCIAL DISTANCING:

***Social distancing* means remaining away from settings where one would gather with others, avoiding mass gatherings, and maintaining a distance of approximately 6 feet (or 2 meters) from others whenever possible, outside of family members.**

During the COVID-19 outbreak:

(55) How much has your time with other people changed compared to how you acted before the COVID-19 outbreak?

Not at all… …An extreme amount

1 2 3 4 5 6 7 8 9 10

(56) How much have you been social distancing during the COVID-19 outbreak?

Not at all… …At all times

1 2 3 4 5 6 7 8 9 10

(57) How stressful has it been for you to maintain social distancing during the COVID-19 outbreak?

Not at all stressful… …Extremely stressful

1 2 3 4 5 6 7 8 9 10

HOPES AND WORRIES:

Please rate how you have felt about the following during the COVID-19 outbreak:

(58) How worried have **you** been about coronavirus (COVID-19)?

Not at all worried… …Extremely worried

1 2 3 4 5 6 7 8 9 10

(59) How worried are you that **you** will be infected with coronavirus (COVID-19)?

Not at all worried… …Extremely worried

1 2 3 4 5 6 7 8 9 10

(60) How worried are you that a **family member** will be infected with coronavirus (COVID-19)?

Not at all worried… …Extremely worried

1 2 3 4 5 6 7 8 9 10

(61) How worried are you that **others around you** will be infected with coronavirus (COVID-19)?

Not at all worried… …Extremely worried

1 2 3 4 5 6 7 8 9 10

(62) During the COVID-19 outbreak, how worried have you been about not being able to afford or access food?

Not at all worried… …Extremely worried

1 2 3 4 5 6 7 8 9 10

(63) How worried have you been about access to important resources such as transportation or housing due to the COVID-19 outbreak?

Not at all worried… …Extremely worried

1 2 3 4 5 6 7 8 9 10

(64) To what degree has the COVID-19 crisis in your area created financial problems for you or your family?

Not at all… …Extremely

1 2 3 4 5 6 7 8 9 10

(65) How much of the day do you actively seek information (read/hear) about coronavirus (COVID-19)?

☐ Not at all ☐ Less than 1 hour ☐ 1-3 hours ☐ 4-6 hours ☐ Greater than 6 hours

(66) Which sources do you consult? Please select all options that apply to you.

☐1 Social media (Facebook, Twitter, Instagram)

☐2 Online journalism

☐3 Television (TV) or radio news

☐4 Word of mouth

☐77 Please specify additional sources you consult: ______________________________

(67) How excessive is your need to seek information on COVID-19?

Not at all excessive… …Extremely excessive

1 2 3 4 5 6 7 8 9 10

(68) To what extent are you avoiding information about COVID-19?

Not at all avoiding… …Complete avoidance

1 2 3 4 5 6 7 8 9 10

(69) How hopeful are you that the coronavirus (COVID-19) problem will end soon?

Not at all hopeful…									…Extremely hopeful
1	2	3	4	5	6	7	8	9	10

(70) How likely is it that the coronavirus (COVID-19) problem will end soon?

Not at all likely…									…Extremely likely
1	2	3	4	5	6	7	8	9	10

(71) How hopeful are you that there will be a vaccine or a cure for coronavirus (COVID-19) within the coming year?

Not at all hopeful…									…Extremely hopeful
1	2	3	4	5	6	7	8	9	10

(72) How likely is it that there will be a vaccine or a cure for coronavirus (COVID-19) within the coming year?

Not at all likely…									…Extremely likely
1	2	3	4	5	6	7	8	9	10

FAMILY & RELATIONSHIPS:

(73) How many people live in your house at the present time (including you)?

I live alone.									
1	2	3	4	5	6	7	8	9	10 or more

(74) Please describe your household members (indicate number):

___ Not applicable, I live alone
___ Number of Child/children below the age of 5
___ Number of Child/children between 5-11
___ Number of Child/children between 12-17
___ Number of Child/children 18 or above
___ Number of Partner/spouse

___ Number of Parent/parents
___ Number of Other relative/relatives
___ Number of Roommate/roommates
___ If not listed above, please specify: ____
___ Someone in my household is pregnant/expecting child (indicate number)

(75) Do you live with any pets?

☐**1** No

☐**2** Yes

(76) Are you experiencing any of the following due to COVID-19 outbreak? Please select all options that apply to you.

☐**1** You lost your job

☐**2** Your salary, hours, or contracts were significantly reduced

☐**3** You are working outside the home as an essential worker

☐**4** You are physically returning to your workplace

☐**5** You are taking on increased childcare duties

☐**6** You are spending increased time to educate or support your child/children's education

☐**7** You have fallen physically ill

☐**8** Family/household member lost their job

☐**9** Family/household member's salary, hours, or contracts were significantly reduced

☐**10** Family/household member or friend fallen physically ill

☐**11** Family/household member with a mental illness is showing increased symptoms

☐**12** Family/household member died of COVID19

☐**13** Family/household member died unrelated to COVID19

☐**14** Someone you know has died of the Coronavirus

☐**15** Someone you know has lost a friend or family member to the Coronavirus

☐**16** None of the above

(77) How have the relationships between members of your family/household been during the COVID-19 outbreak?

Extremely negative…									…Extremely positive
1	2	3	4	5	6	7	8	9	10

(78) During the COVID-19 outbreak, how often did you feel that you lack companionship?

☐1 Hardly ever ☐2 Some of the time ☐3 Often

(79) During the COVID-19 outbreak, how often did you feel left out?

☐1 Hardly ever ☐2 Some of the time ☐3 Often

(80) During the COVID-19 outbreak, how often did you feel isolated from others?

☐1 Hardly ever ☐2 Some of the time ☐3 Often

DAILY ACTIVITIES:

(81) Please check all that apply and rate the extent of your overall engagement in the following activities relative to before the COVID-19 outbreak.

Exercise. Please select all options that apply to you:

☐1 Walking

☐2 Running

☐3 Spinning

☐4 Yoga

☐5 Weights

☐6 Dancing

☐7 Biking

☐8 Hiking

☐77 Please specify additional exercise: ______________________________________

☐78 No, I do not exercise.

(81a) My exercise activity level has been:

Less than usual…				Same as usual					…More than usual
1	2	3	4	5	6	7	8	9	10

(82) Mindfulness. Please select all options that apply to you:

☐1 Meditation

☐2 Deep Breathing

☐3 Body Scan

☐4 Visualization

☐5 Prayer

☐6 Religious/faith service

☐77 Please specify additional mindfulness: ______________________________________

(83) Overall, how much have you been engaging in mindfulness activities:

Less than usual…				Same as usual					…More than usual
1	2	3	4	5	6	7	8	9	10

(84) Hobbies. Please select all options that apply to you:

☐1 Gardening

☐2 Arts/Crafts

☐3 Reading

☐4 Writing

☐5 Watching television (TV)/movies

☐6 Video Games

☐7 Cooking/Baking

☐8 Music

☐77 Please specify additional hobbies: ______________________________________

(85) Overall, how much have you been engaging in these hobbies:

Less than usual…				Same as usual					…More than usual
1	2	3	4	5	6	7	8	9	10

HEALTH CONCERNS:

(86) How has your sleep changed due to the COVID-19 outbreak?

Sleeping much less…				No change					…Sleeping much more
1	2	3	4	5	6	7	8	9	10

(87) How much has your physical activity changed due to the COVID-19 outbreak?

Much less active…				No change					…Much more active
1	2	3	4	5	6	7	8	9	10

FUNCTIONING:

(88) Please rate the extent to which you have experienced **difficulties** in your life due to the COVID-19 outbreak:

I have experienced no difficulties …									…I have experienced extreme difficulties
1	2	3	4	5	6	7	8	9	10

(89) Please rate the degree of **distress** that you have had due to the COVID-19 outbreak:

Not at all distressed…									…Extremely distressed
1	2	3	4	5	6	7	8	9	10

This is the end of the survey. Thank you for your valuable time in completing this survey. If you need to review your responses, go to the previous page(s) before submitting the responses. When you have completed the survey, click on the next page button to submit your responses online. When the survey is submitted, you will see a submission confirmation message.

Please know that because the research study is being done online and your responses will not be monitored in real time, we will not be making contact with you.

Please contact Asian Health & Service Center at designated language specific lines for more information:
English: 503-772-5888
Cantonese: 503-772-5889
Mandarin: 503-772-5890
Korean: 503-772-5891
Vietnamese: 503-772-5892
You can also email at info@ahscpdx.org or to contact your health care provider for information.

In general, if you have any emergent issues, you can call 911 or the SAMHSA National Hotline 1-800-662-HELP (4357) which operates 24/7, 365-day-a-year and provides treatment referral and information service.

Please ask for interpreter services as needed.

Please talk with Collaborator at Asian Health & Service regarding your gift card upon completion of the research study.
