# Supplementary material for: Toward Sustaining Web-Based Senior Center Programming Accessibility With and for Older Adult Immigrants: Community-Based Participatory Research Cross-Sectional Study
Source: Asian Pac Isl Nurs J. 2024 Jan 26;8:e49493. doi: 10.2196/49493 (PMC10858412; doi:10.2196/49493)
Supplement: Multimedia Appendix 3 [file apinj_v8i1e49493_app3.pdf]

Nguyen-Truong CKY, Wuestney KA, Leung H, Chiu C, Park M, Chac C, Fritz RL. Toward sustaining virtual senior center programming accessibility with and for older adult immigrants: a community-based participatory research cross-sectional study. Asian Pac Isl Nurs J. 2024. http://dx.doi.org/10.2196/49493

Multimedia Appendix 3

Table S1. Sociodemographics and background characteristics of older Asian American immigrant adults and Chinese, Korean, and Vietnamese groups.

Variables		Total older Asian American immigrants (n=216)[a]	Subtotal across older Chinese, Korean, and Vietnamese immigrants (n=193)[b]	Chinese immigrants (n=86)[c]	Korean immigrants (n=54)[c]	Vietnamese immigrants (n=53)[c]
**Race and ethnicity, n (%)**						
	Chinese	86 (39.8)	86 (44.6)	86 (100)	0 (0)	0 (0)
	Korean	54 (25)	54 (27.9)	0 (0)	54 (100)	0 (0)
	Vietnamese	53 (24.5)	53 (27.5)	0 (0)	0 (0)	53 (100)
	Taiwanese	15 (6.9)	0 (0)	0 (0)	0 (0)	0 (0)
	Multiracial	7 (3.2)	0 (0)	0 (0)	0 (0)	0 (0)
	Specified Asian race and ethnicity different from above mentioned ones	1 (0.5)	0 (0)	0 (0)	0 (0)	0 (0)
Age (years), mean (SD)		72.7 (6.8)	72.9 (6.9)	72.7 (6.6)	71.7 (7)	74.3 (7)
Age at which the participant moved to the United States to live (years), mean (SD)		43.9 (13.8)	44.9 (13.7)	46 (14.6)	38.7 (11.5)	49.4 (12.3)
**Gender, n (%)**						
	Woman	148 (68.5)	136 (70.5)	62 (72)	39 (72)	35 (66)
	Man	67 (31)	56 (29)	23 (27)	15 (28)	18 (34)
	Specified gender different from abovementioned ones	1 (0.5)	1 (0.5)	1 (1)	0 (0)	0 (0)
**Relationship status, n (%)**						
	Single and never married	10 (4.6)	8 (4.1)	0 (0)	2 (4)	6 (11)
	Married	137 (63.4)	121 (62.7)	65 (76)	28 (52)	28 (53)
	Separated	6 (2.8)	6 (3.1)	0 (0)	0 (0)	6 (11)
	Divorced	20 (9.3)	19 (9.8)	6 (7)	13 (24)	—
	Widowed	43 (19.9)	39 (20.2)	15 (17)	11 (20)	13 (25)
**Education level, n (%)**						

	Less than high school	0 (0)	55 (28.5)	30 (35)	8 (15)	17 (32)
	No school	5 (2.3)	0 (0)	0 (0)	0 (0)	0 (0)
	Some elementary school	7 (3.2)	0 (0)	0 (0)	0 (0)	0 (0)
	Completed elementary school	5 (2.3)	0 (0)	0 (0)	0 (0)	0 (0)
	Some middle school	2 (0.9)	0 (0)	0 (0)	0 (0)	0 (0)
	Completed middle school	15 (6.9)	0 (0)	0 (0)	0 (0)	0 (0)
	Some high school	25 (11.6)	0 (0)	0 (0)	0 (0)	0 (0)
	Graduated high school	74 (34.3)	67 (34.7)	22 (26)	18 (33)	27 (51)
	Had postsecondary education	0 (0)	71 (36.8)[b]	34 (40)	28 (52)	9 (17)
	Some college	24 (11.1)	0 (0)	0 (0)	0 (0)	0 (0)
	Graduated college/professional degree	49 (22.7)	0 (0)	0 (0)	0 (0)	0 (0)
	Some graduate school	3 (1.4)	0 (0)	0 (0)	0 (0)	0 (0)
	Graduated graduate school/professional degree	7 (3.2)	0 (0)	0 (0)	0 (0)	0 (0)
**Employment status, n (%)**						
	Not employed or retired	0 (0)	167 (86.5)	79 (92)	39 (72)	49 (93)
	Not employed	13 (6)	0 (0)	0 (0)	0 (0)	0 (0)
	Retired	175 (81)	0 (0)	0 (0)	0 (0)	0 (0)
	Part-time or full-time work	0 (0)	26 (13.5)[b]	7 (8)	15 (28)	4 (8)
	Part-time work	19 (8.8)	0 (0)	0 (0)	0 (0)	0 (0)
	Full-time work	9 (4.2)	0 (0)	0 (0)	0 (0)	0 (0)
**Type of work (past or present; multiple responses)[d], n (%)**						
	Management, business, science, and arts occupations	68 (31.8)	60 (31.4)	29 (35)	17 (32)	14 (26)
	Service occupations	79 (36.9)	67 (35.1)	28 (33)	27 (50)	12 (23)
	Sales and office occupations	36 (16.8)	31 (16.2)	12 (14)	11 (20)	8 (15)

	Natural resources, construction, and maintenance occupations	13 (6.1)	13 (6.8)	6 (7)	6 (11)	1 (2)
	Production, transportation, and material-moving occupations	37 (17.3)	33 (17.3)	7 (8)	8 (15)	18 (34)
	Household partner or household parent	14 (6.5)	11 (5.8)	6 (7)	3 (6)	2 (4)
	No working history	11 (5.1)	11 (5.8)	5 (6)	1 (2)	5 (9)
**Total annual household income before taxes ($US), n (%)**						
	<15,000	123 (56.9)	117 (60.6)	51 (59)	30 (56)	36 (68)
	15,000-30,000	57 (26.4)	48 (24.9)	21 (24)	18 (33)	9 (17)
	30,001-50,000	20 (9.3)	15 (7.8)	10 (12)	2 (4)	3 (6)
	50,001-60,000	5 (2.3)	4 (2.1)	0 (0)	0 (0)	4 (8)
	60,001-75,000	2 (0.9)	2 (1)	0 (0)	1 (2)	1 (2)
	75,001-100,000	2 (0.9)	2 (1)	0 (0)	2 (4)	0 (0)
	100,001-150,000	1 (0.5)	0 (0)	0 (0)	0 (0)	0 (0)
	≥150,001	2 (0.9)	1 (0.5)	0 (0)	1 (2)	0 (0)
	Not sure	4 (1.9)	4 (2.1)	4 (5)	0 (0)	0 (0)
**Language preference when speaking with a health care provider, n (%)**						
	Chinese Cantonese	48 (22.2)	43 (22.3)	43 (50)	0 (0)	0 (0)
	Chinese Mandarin	49 (22.7)	36 (18.7)	36 (42)	0 (0)	0 (0)
	Korean	43 (19.9)	43 (22.3)	0 (0)	43 (80)	0 (0)
	Vietnamese	53 (24.5)	53 (27.5)	0 (0)	0 (0)	53 (100)
	English	23 (10.6)	18 (9.3)	7 (8)	11 (20)	0 (0)
**Type of health insurance (multiple responses), n (%)**						
	Medicaid and other government insurance	1 (0.5)	1 (0.5)	1 (1)	0 (0)	0 (0)
	Medicaid only	34 (15.7)	30 (15.5)	24 (28)	3 (6)	3 (6)
	Medicare and Medicaid	76 (35.2)	72 (37.3)	21 (24)	24 (44)	27 (51)
	Medicare and other government insurance	1 (0.5)	1 (0.5)	1 (1)	0 (0)	0 (0)
	Medicare only	74 (34.3)	62 (32.1)	32 (37)	16 (30)	14 (26)

	Medicare and private insurance	4 (1.9)	3 (1.6)	0 (0)	2 (4)	1 (2)
	Private health insurance only	21 (9.7)	19 (9.8)	3 (4)	9 (17)	7 (13)
	Specified health insurance different from abovementioned ones	2 (0.9)	2 (1)	2 (2)	0 (0)	0 (0)
	Have no health insurance	3 (1.4)	3 (1.6)	2 (2)	0 (0)	1 (2)
**Have a regular place of care for nonemergency health care services, n (%)**						
	No	3 (1.4)	3 (1.6)	3 (4)	0 (0)	0 (0)
	Not sure	2 (0.9)	1 (0.5)	1 (1)	0 (0)	0 (0)
	Yes	211 (97.7)	189 (97.9)	82 (95)	54 (100)	53 (100)
**Type of regular place of care for nonemergency health care services (multiple responses)[e], n (%)**						
	Private primary care provider office (independent practice or group practice)	94 (44.3)	83 (43.9)	20 (24)	54 (100)	9 (13)
	Medical home	90 (42.5)	84 (44.4)	42 (51)	0 (0)	42 (81)
	Community health service center	14 (6.6)	12 (6.3)	6 (7)	0 (0)	6 (12)
	County health clinic	27 (12.7)	24 (12.7)	22 (27)	0 (0)	2 (4)
	Free health clinic	2 (0.9)	2 (1.1)	1 (1)	0 (0)	1 (2)
	Specified nonemergency health care services different from abovementioned ones	1 (0.5)	1 (0.5)	1 (1)	0 (0)	0 (0)

[a]Responses from participants who identified as Chinese, Korean, Vietnamese, Taiwanese, and multiracial and a participant who specified Asian race and ethnicity different from those listed previously.

[b]Responses from participants who identified as Chinese, Korean, and Vietnamese.

[c]Responses from participants who identified as Chinese, Korean, or Vietnamese.

[d]Overall, 2 missing responses from Chinese group; total sample size=214; subtotal sample size=191; Chinese sample size=84; Korean sample size=54; Vietnamese sample size=53.

[e]Overall, 4 missing responses, of which 3 (75%) were from the Chinese group and 1 (25%) was from the Vietnamese group; total sample size=212; subtotal sample size=189; Chinese sample size=83; Korean sample size=54; Vietnamese sample size=23.
