# Supplementary material for: Toward Sustaining Web-Based Senior Center Programming Accessibility With and for Older Adult Immigrants: Community-Based Participatory Research Cross-Sectional Study
Source: Asian Pac Isl Nurs J. 2024 Jan 26;8:e49493. doi: 10.2196/49493 (PMC10858412; doi:10.2196/49493)
Supplement: Multimedia Appendix 4 [file apinj_v8i1e49493_app4.pdf]

Nguyen-Truong CKY, Wuestney KA, Leung H, Chiu C, Park M, Chac C, Fritz RL. Toward sustaining virtual senior center programming accessibility with and for older adult immigrants: a community-based participatory research cross-sectional study. Asian Pac Isl Nurs J. 2024. http://dx.doi.org/10.2196/49493

Multimedia Appendix 4

Table S2. Psychosocial needs of and effects of the COVID-19 pandemic on older Asian American immigrant adults and Chinese, Korean, and Vietnamese groups.

	Total older Asian American immigrants[a]	Subtotal across older Chinese, Korean, and Vietnamese immigrants[b]	Chinese immigrants[c]	Korean immigrants[c]	Vietnamese immigrants[c]	*P* value	Chi-square (*df*)[d]
**Overall psychosocial health**							
**Has your ability to enjoy things decreased due to the COVID-19 outbreak? (1=not at all decreased to 10=decreased an extreme amount)**						.003[d]	11.5 (2)
Sample size, n	215	192	85	54	53	N/A[e]	N/A
Score, median (IQR)	5 (2-7)	5 (2-7)	5 (4-7)	2 (1-8)	3 (2-7)	N/A	N/A
**How well have you been able to concentrate or focus during the COVID-19 outbreak? (1=not at all to 10=extremely well)**						<.0001[d]	44.7 (2)
Sample size, n	215	192	86	54	52	N/A	N/A
Score, median (IQR)	6 (5-8)	6 (5-8)	5 (4-6)	6 (1-8)	8 (8-9)	N/A	N/A
**Has your ability to solve problems decreased due to the COVID-19 outbreak? (1=not at all decreased to 10=decreased an extreme amount)**						.0865[d]	4.9 (2)
Sample size, n	215	192	85	54	53	N/A	N/A
Score, median (IQR)	4 (1-6)	4 (1.8-6)	5 (3-6)	2 (1-6)	2 (2-7)	N/A	N/A
**Social distancing**							
**How much have you been social distancing during the COVID-19 outbreak? (1=not at all to 10=at all times)**						<.0001[d]	33.6 (2)
Sample size, n	215	192	85	54	53	N/A	N/A
Score, median (IQR)	9 (8-10)	9 (8-10)	8 (6-10)	10 (9-10)	9 (9-10)	N/A	N/A
**How stressful has it been for you to maintain social distancing due to the COVID-19 outbreak? (1=not at all stressful to 10=extremely stressful)**						.0003[d]	16.1 (2)
Sample size, n	214	191	85	54	52	N/A	N/A

		Score, median (IQR)	5 (2-8)	6 (3-8)	5 (1-7)	5 (2-9.8)	7 (5-9)	N/A	N/A
**Worries**									
	**How worried have you been about coronavirus (COVID-19)? (1=not at all worried to 10=extremely worried)**							<.0001[d]	37.9 (2)
		Sample size, n	215	192	85	54	53	N/A	N/A
		Score, median (IQR)	7 (4-8.5)	7 (4-8.2)	7 (4-8)	5 (1-8)	8 (7-9)	N/A	N/A
	**How worried are you that you will be infected with coronavirus (COVID-19)? (1=not at all worried to 10=extremely worried)**							<.0001[d]	75.5 (2)
		Sample size, n	215	192	85	54	53	N/A	N/A
		Score, median (IQR)	7 (3-9)	7 (3-9)	7 (4-8)	2 (1-5)	9 (9-9)	N/A	N/A
	**How worried are you that a family member will be infected with coronavirus (COVID-19)? (1=not at all worried to 10=extremely worried)**							<.0001[d]	55.3 (2)
		Sample size, n	215	192	85	54	53	N/A	N/A
		Score, median (IQR)	8 (5-9)	7 (5-9)	7 (5-8)	4.5 (1-7.8)	9 (9-9)	N/A	N/A
	**How worried are you that others around you will be infected with coronavirus (COVID-19)? (1=not at all worried to 10=extremely worried)**							<.0001[d]	70.1 (2)
		Sample size, n	214	191	84	54	53	N/A	N/A
		Score, median (IQR)	7 (5-9)	7 (5-9)	7 (5-8)	2 (1-6)	9 (9-9)	N/A	N/A
	**During the COVID-19 outbreak, how worried have you been about not being able to afford or access food? (1=not at all worried to 10=extremely worried)**							<.0001[d]	62.6 (2)
		Sample size, n	215	192	85	54	53	N/A	N/A
		Score, median (IQR)	3 (1-6)	3 (1-6)	3 (1-5)	1 (1-2)	7 (5-8)	N/A	N/A
	**How worried have you been about access to important resources such as transportation or housing due to the COVID-19 outbreak? (1=not at all worried to 10=extremely worried)**							<.0001[d]	45.4 (2)
		Sample size, n	215	192	85	54	53	N/A	N/A
		Score, median (IQR)	1 (1-5)	1 (1-5.2)	3 (1-5)	1 (1-1)	6 (1-8)	N/A	N/A
	**To what degree has the COVID-19 crisis in your area created financial problems for you or your family? (1=not at all to 10=extremely)**							.0001[d]	17.7 (2)

Sample size, n	215	192	85	54	53	N/A	N/A
Score, median (IQR)	1 (1-5)	1 (1-5)	3 (1-5)	1 (1-1)	2 (1-8)	N/A	N/A
**Functioning**							
**Please rate the extent to which you have experienced difficulties in your life due to the COVID-19 outbreak (1=I have experienced no difficulties to 10=I have experienced extreme difficulties)**						<.0001[d]	51 (2)
Score, median (IQR)	5 (1-6)	5 (1-6)	5 (2-6)	1 (1-3)	6 (5-7)	N/A	N/A
Score, median (IQR)	5 (1-6)	5 (1-6)	5 (2-6)	1 (1-3)	6 (5-7)	N/A	N/A
**Please rate the degree of distress that you have had due to the COVID-19 outbreak (1=not at all distressed to 10=extremely distressed)**						<.0001[d]	22.1 (2)
Sample size, n	215	192	85	54	53	N/A	N/A
Score, median (IQR)	5 (2-6.5)	5 (3-7)	5 (2-6)	4.5 (1-6.8)	7 (5-7)	N/A	N/A
**Family and relationships**							
**Are you experiencing any of the following due to COVID-19 outbreak?[f]**							
Sample size, n	210	187	82	53	53	N/A	N/A
**You are working outside the home as an essential worker, n (%)**						.002[g]	N/A
No	202 (96.2)	182 (96.8)	82 (100)	47 (89)	53 (100)	N/A	N/A
Yes	8 (3.8)	6 (3.2)	0 (0)	6 (11)	0 (0)	N/A	N/A
**You are physically returning to your workplace, n (%)**						.4978[g]	N/A
No	207 (98.6)	186 (98.9)	80 (98)	53 (100)	53 (100)	N/A	N/A
Yes	3 (1.4)	2 (1.1)	2 (2)	0 (0)	0 (0)	N/A	N/A
**You lost your job, n (%)**						.041[g]	N/A
No	205 (97.6)	185 (98.4)	82 (100)	50 (94)	53 (100)	N/A	N/A
Yes	5 (2.4)	3 (1.6)	0 (0)	3 (6)	0 (0)	N/A	N/A
**Your salary, hours, or contracts were significantly reduced, n (%)**						.0465[g]	N/A
No	195 (92.9)	175 (93.1)	79 (96)	45 (85)	51 (96)	N/A	N/A
Yes	15 (7.1)	13 (6.9)	3 (4)	8 (15)	2 (4)	N/A	N/A
**Family/household member lost their job, n (%)**						.0975[f]	N/A
No	185 (88.1)	165 (87.8)	74 (90)	49 (93)	42 (79)	N/A	N/A
Yes	25 (11.9)	23 (12.2)	8 (10)	4 (8)	11 (21)	N/A	N/A
**Family/household member's salary, hours, or contracts were significantly reduced, n (%)**						.0005[g]	N/A
No	183 (87.1)	163 (86.7)	80 (98)	45 (85)	38 (72)	N/A	N/A

Yes	27 (12.9)	25 (13.3)	2 (2)	8 (15)	15 (28)	N/A	N/A
**You have fallen physically ill, n (%)**						.02[g]	N/A
No	191 (91)	173 (92)	73 (89)	47 (89)	53 (100)	N/A	N/A
Yes	19 (9)	15 (8)	9 (11)	6 (11)	0 (0)	N/A	N/A
**Family/household member or friend fallen physically ill, n (%)**						.0005[g]	N/A
No	192 (91.4)	171 (91)	75 (92)	43 (81)	53 (100)	N/A	N/A
Yes	18 (8.6)	17 (9)	7 (9)	10 (19)	0 (0)	N/A	N/A
**Family/household member with a mental illness is showing increased symptoms, n (%)**						.1634[g]	N/A
No	208 (99)	186 (98.9)	82 (100)	51 (96)	53 (100)	N/A	N/A
Yes	2 (1)	2 (1.1)	0 (0)	2 (4)	0 (0)	N/A	N/A
**Family/household member died unrelated to COVID-19, n (%)**						.1999[g]	N/A
No	207 (98.6)	185 (98.4)	82 (100)	52 (98)	51 (96)	N/A	N/A
Yes	3 (1.4)	3 (1.6)	0 (0)	1 (2)	2 (4)	N/A	N/A
**Someone you know has died of the coronavirus, n (%)**						.2439[g]	N/A
No	161 (76.7)	144 (76.6)	66 (81)	36 (68)	42 (79)	N/A	N/A
Yes	49 (23.3)	44 (23.4)	16 (20)	17 (32)	11 (21)	N/A	N/A
**Someone you know has lost a friend or family member to the coronavirus, n (%)**						.021[g]	N/A
No	167 (79.5)	146 (77.7)	71 (87)	40 (76)	35 (66)	N/A	N/A
Yes	43 (20.5)	42 (22.3)	11 (13)	13 (25)	18 (34)	N/A	N/A
**How many people live in your house at the present time (including you)?[h] (1=live alone, 2=2 people, 3=3 people, 4=4 people, 5=5 people, 6=6 people, 7=7 people, 8=8 people, 9=9 people, and 10=≥10 people; multiple responses)**						.0064[d]	10.1 (2)
Sample size, n	215	192	85	54	53	N/A	N/A
Score, median (IQR)	2 (1-2)	2 (1-2)	2 (2-2)	2 (1-2)	2 (1-3)	N/A	N/A
Live alone, n (%)	64 (29.8)	59 (30.7)	19 (22)	24 (44)	16 (30)	N/A	N/A
2 people, n (%)	112 (52.1)	99 (51.6)	51 (60)	27 (50)	21 (40)	N/A	N/A
3 people, n (%)	19 (8.8)	15 (7.8)	6 (7)	0 (0)	9 (17)	N/A	N/A
4 people, n (%)	9 (4.2)	9 (4.7)	2 (2)	2 (4)	5 (9)	N/A	N/A
5 people, n (%)	5 (2.3)	5 (2.6)	4 (5)	0 (0)	1 (2)	N/A	N/A
6 people, n (%)	2 (0.9)	1 (0.5)	0 (0)	1 (2)	0 (0)	N/A	N/A
7 people, n (%)	3 (1.4)	3 (1.6)	3 (3.5)	0 (0)	0 (0)	N/A	N/A
8 people, n (%)	0 (0)	0 (0)	0 (0)	0 (0)	0 (0)	N/A	N/A
9 people, n (%)	0 (0)	0 (0)	0 (0)	0 (0)	0 (0)	N/A	N/A

		≥10 people, n (%)	1 (0.5)	1 (0.5)	0 (0)	0 (0)	1 (2)	N/A	N/A
	**How have the relationships between members of your family/household been during the COVID-19 outbreak? (1=extremely negative to 10=extremely positive)**							<.0001[d]	33.2 (2)
		Sample size, n	214	191	85	54	52	N/A	N/A
		Score, median (IQR)	8 (5-9)	8 (5-9)	6 (5-8)	9 (7.2-10)	9 (7.8-9.2)	N/A	N/A
	**3-item loneliness[i] (1=hardly ever, 2=some of the time, and 3=often)**							.0745[d]	5.2 (2)
		Sample size, n	215	192	85	54	53	N/A	N/A
		Score, median (IQR)	4 (3-6)	4 (3-6)	4 (3-5)	4 (3-5)	6 (3-6)	N/A	N/A
	**Do you live with any pets?[j]**							.0325[g]	N/A
		Sample size, n	205	182	84	51	48	N/A	N/A
		No, n (%)	184 (89.8)	165 (90.2)	79 (94)	41 (80)	45 (94)	N/A	N/A
		Yes, n (%)	21 (10.2)	18 (9.8)	5 (6)	10 (20)	3 (6)	N/A	N/A
**Daily activities**									
	**How much has your physical activity changed due to the COVID-19 outbreak? (1=much less active, 5=no change, and 10=much more active)**							.0041[d]	11 (2)
		Sample size, n	215	192	85	54	53	N/A	N/A
		Score, median (IQR)	5 (3-5)	5 (3-5)	4 (3-5)	5 (4-5)	5 (4-5)	N/A	N/A
	**Exercise activities participants engage in[k] (multiple responses)**								
		Sample size, n	215	192	85	54	53	N/A	N/A
		Walking, n (%)	175 (81.4)	157 (81.8)	63 (74.1)	43 (80)	51 (96)	N/A	N/A
		Running, n (%)	9 (4.2)	8 (4.2)	6 (7.1)	2 (4)	0 (0)	N/A	N/A
		Spinning, n (%)	6 (2.8)	5 (2.6)	2 (2.4)	3 (6)	0 (0)	N/A	N/A
		Yoga, n (%)	9 (4.2)	8 (4.2)	3 (3.5)	3 (6)	2 (4)	N/A	N/A
		Weights, n (%)	7 (3.3)	6 (3.1)	1 (1.2)	4 (7)	1 (2)	N/A	N/A
		Dancing, n (%)	21 (9.8)	17 (8.9)	15 (17.6)	1 (2)	1 (2)	N/A	N/A
		Biking, n (%)	4 (1.9)	3 (1.6)	2 (2.4)	1 (2)	0 (0)	N/A	N/A
		Hiking, n (%)	14 (6.5)	10 (5.2)	4 (4.7)	6 (11)	0 (0)	N/A	N/A
		Specified exercise is not listed, n (%)	56 (26)	49 (25.5)	34 (40)	11 (20)	4 (8)	N/A	N/A
		No I do not exercise, n (%)	14 (6.5)	12 (6.2)	5 (5.9)	5 (9)	2 (4)	N/A	N/A

**My exercise activity level has been... (1=less than usual, 5=same as usual, and 10=more than usual)**						<.0001[d]	20.2 (2)
Sample size, n	215	192	85	54	53	N/A	N/A
Score, median (IQR)	5 (3-5)	5 (3-5)	4 (3-5)	5 (3-5)	5 (4-5)	N/A	N/A
**Mindfulness activities participants engage in[l] (multiple responses)**						N/A	N/A
Sample size, n	201	178	72	54	52	N/A	N/A
Meditation, n (%)	49 (26.9)	46 (27.5)	13 (21)	14 (26)	19 (37)	N/A	N/A
Deep breathing, n (%)	22 (12.1)	19 (11.4)	12 (20)	4 (7)	3 (6)	N/A	N/A
Body scan, n (%)	3 (1.6)	3 (1.8)	3 (5)	0 (0)	0 (0)	N/A	N/A
Visualization, n (%)	10 (5.5)	10 (6)	10 (16)	0 (0)	0 (0)	N/A	N/A
Prayer, n (%)	99 (54.4)	91 (54.5)	16 (26)	36 (67)	39 (75)	N/A	N/A
Religious/faith service, n (%)	92 (50.5)	83 (49.7)	19 (31)	34 (63)	30 (58)	N/A	N/A
Specified activity different from abovementioned ones, n (%)	26 (14.3)	24 (14.4)	16 (26)	8 (15)	0 (0)	N/A	N/A
**Overall, how much have you been engaging in mindfulness activities (1=less than usual, 5=same as usual, and 10=more than usual)**						.005[d]	10.6 (2)
Sample size, n	201	178	72	54	52	N/A	N/A
Score, median (IQR)	5 (5-5)	5 (5-5)	5 (5-5)	5 (5-6.5)	5 (5-7)	N/A	N/A
**Hobbies participants engage in[m] (multiple responses)**						N/A	N/A
Sample size, n	215	192	85	54	53	N/A	N/A
Gardening, n (%)	75 (34.9)	60 (31.2)	28 (33)	14 (26)	18 (34)	N/A	N/A
Arts/crafts, n (%)	19 (8.8)	16 (8.3)	9 (11)	7 (13)	0 (0)	N/A	N/A
Reading, n (%)	75 (34.9)	69 (35.9)	22 (26)	19 (35)	28 (53)	N/A	N/A
Writing, n (%)	22 (10.2)	21 (10.9)	7 (8)	11 (20)	3 (6)	N/A	N/A

		Watching television/movies, n (%)	122 (56.7)	106 (55.2)	47 (55)	26 (48)	33 (62)	N/A	N/A
		Video games, n (%)	2 (0.9)	2 (1)	1 (1)	1 (2)	0 (0)	N/A	N/A
		Cooking/baking, n (%)	77 (35.8)	69 (35.9)	27 (32)	16 (30)	26 (50)	N/A	N/A
		Music, n (%)	49 (22.8)	45 (23.4)	16 (19)	15 (28)	14 (26)	N/A	N/A
		Specified hobbies are not listed, n (%)	51 (23.7)	43 (22.4)	32 (38)	7 (13)	4 (8)	N/A	N/A
	**Overall, how much have you been engaging in these hobbies (1=less than usual, 5=same as usual, and 10=more than usual)**							<.0001[d]	26.6 (2)
		Sample size, n	215	192	85	54	53	N/A	N/A
		Score, median (IQR)	5 (5-6)	5 (5-6)	5 (5-5)	5 (5-5)	6 (5-7)	N/A	N/A

[a]Responses from participants who identified as Chinese, Korean, Vietnamese, Taiwanese, and multiracial and a participant who specified Asian race and ethnicity different from those listed previously.

[b]Responses from participants who identified as Chinese, Korean, and Vietnamese.

[c]Responses from participants who identified as Chinese, Korean, or Vietnamese.

[d]Kruskal-Wallis rank sum test.

[e]N/A: not applicable.

[f]Overall, 6 missing responses, of which 1 (16%) was from the Asian American group, 4 (68%) were from the Chinese group and 1 (16%) was from the Korean group.

[g]Fisher exact test.

[h]A response was missing from the Chinese group.

[i]Score of 3-item loneliness: "During the COVID-19 outbreak, how often did you feel that you lack companionship?" "During the COVID-19 outbreak, how often did you feel left out?" and "During the COVID-19 outbreak, how often did you feel isolated from others?"

[j]Overall, 11 missing responses, of which 1 (10%) was from the Asian American group, 2 (18%) were from the Chinese group, 3 (27%) were from the Korean group, and 5 (45%) were from the Vietnamese group.

[k]A response was missing from the Chinese group.

[l]Overall, 34 missing responses, of which 8 (23%) was from the Asian American group, 25 (74%) were from the Chinese group and 1 (3%) was from the Vietnamese group.

[m]A response was missing from the Chinese group.
